# Supplementary material for: Genome-wide association analyses identify distinct genetic architectures for early-onset and late-onset depression
Source: Nat Genet. 2025 Nov 13;57(12):2972–9. doi: 10.1038/s41588-025-02396-8 (PMC12695632; doi:10.1038/s41588-025-02396-8)
Supplement: Supplementary file 1 — Supplementary Figs. 1–8 and Supplementary Notes (Methods and Discussion). [file 41588_2025_2396_MOESM1_ESM.pdf]

nature genetics

Supplementary information

https://doi.org/10.1038/s41588-025-02396-8

# Genome-wide association analyses identify distinct genetic architectures for early-onset and late-onset depression

In the format provided by the authors and unedited

**Supplementary Information**

# Genome-wide association analyses identify distinct genetic architectures for early- and late-onset depression

## Table of Contents

*Supplementary Notes* ........ *2*

Supplementary Methods ........ 2

Supplementary Discussion ........ 5

Supplementary Figures ........ 7

# Supplementary Notes

## Supplementary Methods

**Cohorts**

Denmark

Genetic data used in the Danish cohorts came from The Integrative Psychiatric Research Consortium (iPSYCH) cohort, described previously[1,2]. In brief, the study base for the population-based cohort and all MDD case groups includes singletons born in Denmark between May 1, 1981, and December 31, 2008, who were alive and resided in Denmark on their one-year birthday, and who have a known mother (N = 1,657,449 persons). Following quality control that censored individuals of non-European origins[3], 31,291 individuals were selected as MDD cases, with 26,534 eoMDD cases, and 39,626 individuals selected as controls. No cases were loMDD since the iPSYCH samples were relatively young.

The iPSYCH cohort consists of two separate studies, hereafter referred to as iPSYCH2012 and iPSYCH2015. iPSYCH2012 genotyping was performed using DNA from dried blood spots obtained from the Danish neonatal screening biobank with the Infinium PsychChip Array v1.0 (Illumina, San Diego CA, USA) at The Broad Institute, Boston MA, USA. iPSYCH2015 is an extension of iPSYCH2012, where genotyping was performed similarly to iPSYCH2012 but using the Illumina Global Screening Array v2.0 (Illumina, San Diego CA, USA) at Statens Serum Institut, Copenhagen DK. A total of 80,876 individuals were genotyped at 251,551 SNPs for iPSYCH2012, and 48,974 individuals were genotyped at 450,445 SNPs for iPSYCH2015. Both cohorts were imputed using BEAGLE5.1 and the Haplotype Reference Consortium (HRC, r1.1) as the reference[3]. SNPs were excluded if they were missing in excess of 5% of the samples, a minor allele frequency threshold was set to 0.001, and the Hardy-Weinberg cutoff was set to $p$<1x10^-6. Following imputation quality control[3], 11,530,005 SNPs were present in iPSYCH2012 and 12,044,511 SNPs were present in iPSYCH2015. Sex was inferred using heterozygosity on chromosome X; below 20% in males; above 20% in females. Sex obtained from genotyping was compared to the sex recorded in the Danish Civil Registration System and samples with a mismatch were excluded[2]. All electronic health care records were obtained using the Danish Civil Registration System, established in 1968, which links data among registers using a unique identification number assigned to all Danish residents. Information on mental diseases was obtained from the Danish Psychiatric Central Research Register, which contains information on all admissions to psychiatric inpatient facilities since 1969 and visits to outpatient wards since 1995[2].

Norway

The Norwegian Mother, Father and Child Cohort Study (MoBa) is a population-based pregnancy cohort study conducted by the Norwegian Institute of Public Health. Participants were recruited from all over Norway from 1999-2008. The women consented to participation in 41% of the pregnancies. The cohort includes approximately 114,500 children, 95,200 mothers and 75,200 fathers. The current study is based on version 12 of the quality-assured data files released for research in 2021-07-14. The establishment of MoBa and initial data collection was based on a license from the Norwegian Data Protection Agency and approval from The Regional Committees for Medical and Health Research Ethics. The MoBa cohort is currently regulated by the Norwegian Health Registry Act. The current study was approved by The Regional Committees for Medical and Health Research Ethics (REK

2016/1226). In the current study, we used a subsample of 207,569 (73%) individuals that were genotyped. Blood samples were obtained from both parents during pregnancy and from mothers and children (umbilical cord) at birth.

MoBa was genotyped in different batches as part of several research projects and was quality-controlled using the MoBaPsychGen genotype QC pipeline (https://github.com/psychgen/MoBaPsychGen-QC-pipeline), which is specifically developed to handle the complex relatedness in MoBa participants. This pipeline includes standard QC protocols (comparable to e.g. the Ricopili imputation pipeline). Briefly, SNPs with minor allele frequency < 0.5%, call rate < 98%, or Hardy Weinberg Equilibrium at p-value < 1x10^-6, and individuals outside three standard deviations from the mean heterozygosity or with discordant sex were removed. Phasing and imputations were performed in chunks of up to 5000 individuals using the Haplotype Reference Consortium release 1.1 as reference. Further details are provided elsewhere[4].

Through the personal identification number, which is assigned at birth, MoBa participants have been linked to several health registers, such as the Norwegian National Patient Register (NNPR)[5]. The NNPR contains information on all registered contacts with the Norwegian specialist health system since 2008, including in- and out-patient services.

Sweden

We included several Swedish cohorts that had genetic data and phenotypic data on MDD. For both cases and controls in these cohorts, the exclusion criteria were any diagnosis of bipolar disorder, schizoaffective disorder, or schizophrenia.

*PREFECT* vs *ANGI:* The PREFECT sample constituted N=1,796 cases who had a clinical diagnosis of MDD and were treated for a major depressive episode with electroconvulsive therapy (ECT)[6]. Participants were recruited through the Swedish National Quality Register for ECT between 2013 and 2017. For the case-control GWAS, PREFECT cases were compared with 3,624 controls selected from the LifeGene study[7], a population-based sample that was part of a previous study on eating disorders ("ANGI")[6]. In addition to the standard exclusions, controls with a history of eating disorders were excluded in this study. Due to the predominance of eating disorders in females, this cohort was largely female (82%). All participants were genotyped using Illumina GSA-MD SNP arrays (v1). Genotype data were cleaned using the Ricopoli pipeline, and imputed to the HRC r1.1 reference panel[6]. For the analyses of early-onset MDD, we used controls from STAGE instead (see below) since it provided a larger control sample.

*iCBT* vs *LifeGene:* iCBT cases were individuals diagnosed with MDD who were treated at the Internet Psychiatry Clinic in Stockholm county (N=906)[8,9]. In addition to the standard exclusions, severe and suicidal cases and individuals presenting with alcohol or drug abuse were excluded from this study. Controls were again retrieved from LifeGene (non-overlapping with the PREFECT controls; N=4,717) and had no history of MDD (self-reported). Roughly half of the cohort consisted of women (52%). Cases were genotyped using the Infinium GSA 1.0 BeadArray. The PGC Ricopili pipeline was used for QC and imputed to 1000 Genomes European reference data (phase 1, version 3).

Study of Twin Adults: Genes and Environment (*STAGE):* This cohort consisted of those born between 1959 and 1985 from the Swedish Twin Registry[10]. Cases were identified using the National Patient Registry (N cases=424, N controls=9,615). The numbers of eoMDD and loMDD cases were too low to warrant GWAS of the subtypes. More than half of the cohort were women (60%). STAGE

participants were genotyped with the Illumina GSA BeadChip. Data were again QC-ed using the PGC pipeline and imputed to HRC r1.1 and cleaned post-imputation similar to the other cohorts.

*BASIC:* The Biology, Affect, Stress, Imaging and Cognition (BASIC) study recruited 5,492 pregnant women from the Uppsala Hospital University (Uppsala, Sweden) and followed them and their children from pregnancy to one year postpartum between 2012 and 2019[11]. There were 1,003 cases of MDD in the years surrounding pregnancy, and 1,854 controls. MDD was measured using the Mini International Neuropsychiatric Interview (MINI) and clinical questionnaires, and in some cases confirmed using health records. The genotyping bead chip was the Illumina GSA-MD 24v2. QC and imputation procedures followed the PGC Ricopili pipeline.

Finland

FinnGen is a research project combining genomic data from nine Finnish biobanks and ICD-coded diagnoses from electronic health records consisting of several national health registries (https://www.finngen.fi/en)[12]. Data from participants recruited for FinnGen specifically as well as from older disease-specific studies (legacy samples) are included. For this study, participants with Finnish ancestry of data release 10 (N = 413,718) were phenotyped for MDD using inpatient and outpatient specialized health care (data available from 1964 and 1998 onwards, respectively), primary health care (from 2011), cause of death (from 1969) and drug imbursement registers (from 1964). After applying exclusion criteria, we identified 56,661 cases with lifetime MDD, among which 10,031 and 22,029 cases were eoMDD and loMDD, respectively. After matching by sex and age, twice as many controls as cases were available. FinnGen participants were genotyped using the FinnGen ThermoFisher Axiom custom array. Imputation was conducted against a Finnish ancestry-specific reference panel SiSu v4.2 (https://www.sisuproject.fi/) computed from whole-genome sequenced samples of 10,490 Finnish individuals. For QC procedures see previously published work[12].

Estonia

Estonian Biobank (EstBB) is a population-based cohort with a variety of health-related phenotypic data for each participant[13]. Diagnoses (ICD-10 codes) and drug prescription data (ATC codes, prescription status and purchase date) are available for all participants. In addition, a Mental Health online Survey (MHoS) was conducted in 2021 capturing lifestyle and mental health symptoms (N=86,000)[13]. The Core Facility of Genomics, Institute of Genomics, University of Tartu genotyped the DNA samples using Illumina Global Screening Arrays (GSAv1.0, GSAv2.0, GSAv2.0_EST). Individuals with call-rate < 95% or mismatched sex in phenotype data were excluded. Variant filtering was done by call rate <95% and HWE p-value $<1e^{-4}$ (autosomal variants only). After QC, 202,910 samples were available for imputation. We used an Estonian ancestry-specific reference panel computed from 2,695 whole-genome sequenced samples of Estonian individuals for imputation. Then, variants with MAF <1% and indels were removed. After applying exclusion criteria for other psychiatric disorders (schizoaffective disorder, bipolar disorder, and schizophrenia), 49,950 MDD cases remained with 100,660 controls, of which 8,791 eoMDD and 14,656 loMDD cases were identified. For case-control matching sex and year of birth were used to find the most suitable controls for every case. This research was conducted under ethical approvals 1.1-12/3455 and 1.1-12/2860 from the Estonian Committee on Bioethics and Human Research, using data according to the release application nr 6-7/GI/32596 (T37) from the Estonian Biobank.

United Kingdom

UK Biobank data were used for comparison and investigating generalizability. This resource has been extensively mined for research and has been described in detail elsewhere[14]. The UK Biobank contains biological, survey, and register data from up to 500,000 United Kingdom residents aged 40-69. To identify MDD, we used linked electronic health records from hospital inpatient admissions (data field 41270; relying on the same ICD-10 codes as for the other cohorts), which contains data from 1981, 1997 or 1998 (for Scotland, England, and Wales, respectively) to 2022 covering up to 446,996 UK Biobank participants. The collection of healthcare data is less standardized than in the Nordic countries, due to different systems within the countries in the UK, and data quality varies across measurement years (UK-Biobank data resource 138483[15]). For that reason, age at first diagnosis could not reliably be extracted from the health records. For the eoMDD phenotype, we relied on self-reported age at first episode ('How old were you the FIRST time you had a period of two weeks like this', referring to 'feelings of depression or loss of interest'; data field 20433). Patients with an ICD10 diagnosis of schizoaffective disorder, bipolar disorder, or schizophrenia were excluded. We used the genetic data imputed to the HRC reference panel as provided by the UK Biobank (resource 157020), and applied quality control with minor allele frequency > 0.01, genotype and individual call rate >95%, and HWE $p > 1 \times 10^{-6}$. The final samples included in the GWAS were 25,162 MDD cases, including 3,402 eoMDD and 9,084 loMDD, as well as 431,658 controls.

## Supplementary Discussion

In summary, we conducted a trans-Nordic GWAS of two clinically important MDD subtypes based on age of first diagnosis–eoMDD and loMDD–to identify potential genetic heterogeneity in MDD. Our findings indicate that eoMDD and loMDD have partially distinct genetic signatures which were related to differences in their associated comorbidities. Evidence of a partial genetic overlap is based on the difference in their SNP-based heritability (twice as large for eoMDD as for loMDD), suggestive evidence for a lower polygenicity in eoMDD, and their moderate genetic correlation ($r_g$=0.58). Moreover, we identified genetic loci differentially associated with early- and late-onset MDD.

We also provided evidence for differences in the underlying biological underpinning of these two MDD subtypes as shown by the specific enrichment of fetal brain tissues in the eoMDD genetic signal. This suggests a link between brain development processes and eoMDD, a hypothesis further supported by the genetic overlap between eoMDD and the neurodevelopmental disorders of ASD and ADHD.

The genetic associations between eoMDD, loMDD, and somatic comorbidities confirm the role of shared genetics in these comorbidities. However, we also detected some differences between eoMDD and loMDD, suggesting variability in genetic links to comorbid conditions. Previous studies have indicated that MDD increases the risk for CVD (e.g., coronary artery disease, peripheral artery disease, heart failure, and stroke) and that genetics play a role in the MDD-CVD comorbidity[16]. Our Genomic SEM and PheWAS analyses reveal that the associations with heart failure and stroke may be primarily driven by the eoMDD subtype, emphasizing the importance of MDD subtyping in the clinical management of MDD patients. On the other hand, while the link between MDD and Alzheimer's disease is well established[17,18], previous research did not identify strong shared genetic factors[19]. Similarly, we did not find significant genetic associations in either eoMDD or loMDD. While our subtype GWAS may have lower statistical power, the findings suggest that the magnitude of overall genetic associations between MDD and Alzheimer's disease may be low.

A key strength of this work is the use of decades-long health care records for treated MDD across multiple Nordic countries given similarities in healthcare organization and near-universal healthcare access[20]. Our harmonized approach maximizes similarity in samples and phenotype definitions. However, this approach to ascertain cases mostly from specialist care may impact the findings. For example, the probability of specialist identification of MDD could vary over calendar time, in the presence of comorbid psychiatric and somatic disorders, positive or negative family history, or in local referrable practice[10]. Specifically, eoMDD cases may require specialist care due to co-occurring childhood-onset conditions (e.g., ADHD or autism), while the likelihood of loMDD may be increased by somatic comorbidities common in older age. Our inclusion of birth year as a covariate in all the analyses accounts at least partly for these potential biases. More importantly, the cohorts that had both primary and specialist care (FinnGen, EstBB) showed high genetic correlations with those from specialist diagnosis only, and our findings are consistent with the UK Biobank self-reported symptom data that showed a similar pattern of genetic correlations between eoMDD/loMDD and other psychiatric disorders and related traits[21]. Another limitation is the reliance on age at diagnosis as a proxy for age at onset. Despite the high genetic overlap between these two phenotypes[22], we acknowledge the individual variation in the time gap from symptom onset to receiving a diagnosis may impact our results. We did not apply a unified minimum follow-up restriction across participating cohorts. This would mean that, for the youngest, the follow-up time may be limited to observe a diagnostic conversion to bipolar disorder or schizophrenia. Future research is needed to establish generalizability outside European ancestry populations.

## References:


1. Bybjerg-Grauholm, J. et al. The iPSYCH2015 Case-Cohort sample: updated directions for unravelling genetic and environmental architectures of severe mental disorders. *medRxiv* https://doi.org/10.1101/2020.11.30.20237768 (2020).
2. Pedersen, C. B. *et al.* The iPSYCH2012 case-cohort sample: new directions for unravelling genetic and environmental architectures of severe mental disorders. *Mol Psychiatry* **23**, 6–14 (2018).
3. Appadurai, V. *et al.* Accuracy of haplotype estimation and whole genome imputation affects complex trait analyses in complex biobanks. *Commun Biol* **6**, 101 (2023).
4. Corfield E. C. *et al.* The Norwegian Mother, Father, and Child Cohort Study (MoBa) genotyping data resource: MoBaPsychGen pipeline v.1. *bioRxiv* https://doi.org/10.1101/2022.06.23.496289 (2022).
5. Magnus, P. *et al.* Cohort profile: The Norwegian Mother and Child Cohort Study (MoBa). *International Journal of Epidemiology* **35**, 1146–1150 (2006).

6. Clements, C. C. *et al.* Genome-wide association study of patients with a severe major depressive episode treated with electroconvulsive therapy. *Mol Psychiatry* **26**, 2429–2439 (2021).
7. Almqvist, C. *et al.* LifeGene--a large prospective population-based study of global relevance. *Eur J Epidemiol* **26**, 67–77 (2011).
8. Andersson, E. *et al.* Genetics of response to cognitive behavior therapy in adults with major depression: a preliminary report. *Molecular Psychiatry* **24**, 484–490 (2019).
9. Boberg, J. *et al.* Swedish multimodal cohort of patients with anxiety or depression treated with internet-delivered psychotherapy (MULTI-PSYCH). *BMJ Open* **13**, e069427 (2023).
10. Zagai, U., Lichtenstein, P., Pedersen, N. L. & Magnusson, P. K. E. The Swedish Twin Registry: Content and Management as a Research Infrastructure. *Twin Research and Human Genetics* **22**, 672–680 (2019).
11. Axfors, C. *et al.* Cohort profile: the Biology, Affect, Stress, Imaging and Cognition (BASIC) study on perinatal depression in a population-based Swedish cohort. *BMJ Open* **9**, e031514 (2019).
12. Kurki, M. I. *et al.* FinnGen provides genetic insights from a well-phenotyped isolated population. *Nature* **613**, 508–518 (2023).
13. Ojalo, T. *et al.* Cohort Profile Update: Mental Health Online Survey in the Estonian Biobank (EstBB MHoS). *Int J Epidemiol* **53**, dyae017 (2024).
14. Sudlow, C. *et al.* UK Biobank: An Open Access Resource for Identifying the Causes of a Wide Range of Complex Diseases of Middle and Old Age. *PLOS Medicine* **12**, e1001779 (2015).
15. Wilkinson, T. *et al.* Identifying dementia outcomes in UK Biobank: a validation study of primary care, hospital admissions and mortality data. *Eur J Epidemiol* **34**, 557–565 (2019).

16. Bergstedt, J. *et al.* Distinct biological signature and modifiable risk factors underlie the comorbidity between major depressive disorder and cardiovascular disease. *Nat Cardiovasc Res* **3**, 754–769 (2024).
17. Chow, Y. Y., Verdonschot, M., McEvoy, C. T. & Peeters, G. Associations between depression and cognition, mild cognitive impairment and dementia in persons with diabetes mellitus: A systematic review and meta-analysis. *Diabetes Res Clin Pract* **185**, 109227 (2022).
18. Huang, Y.-Y., Gan, Y.-H., Yang, L., Cheng, W. & Yu, J.-T. Depression in Alzheimer's Disease: Epidemiology, Mechanisms, and Treatment. *Biol Psychiatry* **95**, 992–1005 (2024).
19. Wightman, D. P. *et al.* A genome-wide association study with 1,126,563 individuals identifies new risk loci for Alzheimer's disease. *Nat Genet* **53**, 1276–1282 (2021).
20. Pasman, J. A. *et al.* Epidemiological overview of major depressive disorder in Scandinavia using nationwide registers. *The Lancet Regional Health - Europe* **29**, 100621 (2023).
21. Nguyen, T.-D. *et al.* Genetic heterogeneity and subtypes of major depression. *Mol Psychiatry* **27**, 1667–1675 (2022).
22. Harder, A. *et al.* Genetics of age-at-onset in major depression. *Transl Psychiatry* **12**, 124 (2022).

## Supplementary Figures

# Supplementary Figures

**Table of contents**

Supplementary Figure 1. Genetic correlations
Supplementary Figure 2. Locus zoom plots for eoMDD
Supplementary Figure 3a. SNP lookup in UK-Biobank
Supplementary Figure 3b. SNP lookup in most recent GWAS on broad MDD (Als et al., 2023)
Supplementary Figure 4. Enrichments of SNP heritability in annotations
Supplementary Figure 5. Genetic correlation heatmap
Supplementary Figure 6. Genetic correlation bar plot with broad MDD
Supplementary Figure 7. PRS for the meta-analysis and individual cohorts.
Supplementary Figure 8a. eoMDD PGS-PheWAS results
Supplementary Figure 8b. loMDD PGS-PheWAS results

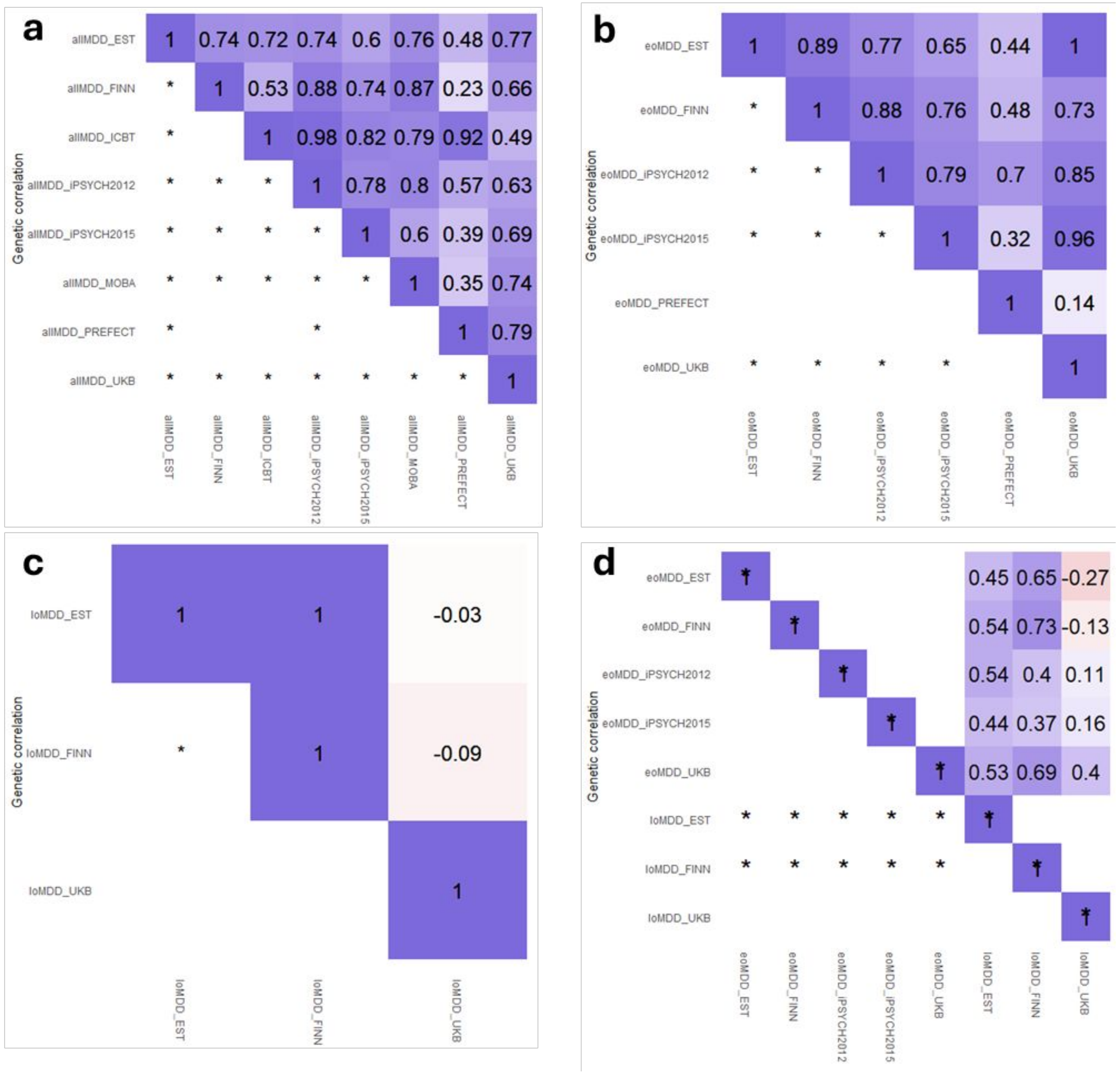


**Supplementary Figure 1.** Genetic correlations between cohorts in the study. a) all MDD. b) Early-onset MDD. c) Late-onset MDD. d) Early-onset MDD to Late-onset MDD. The upper triangle contains the correlations, with shade indicating the strength of the correlation. The lower triangle indicates if the correlation was nominally significant at $p<.05$ (indicated with *).

| | |
|---|---|
| top lead SNP | rs272811 |
| Chrom | 1 |
| BP | 36634414 |
| P-value | 3.597e-08 |
| #Ind. Sig. SNPs | 1 |
| #lead SNPs | 1 |
| SNPs within LD | 40 |
| GWAS SNPs within LD | 31 |

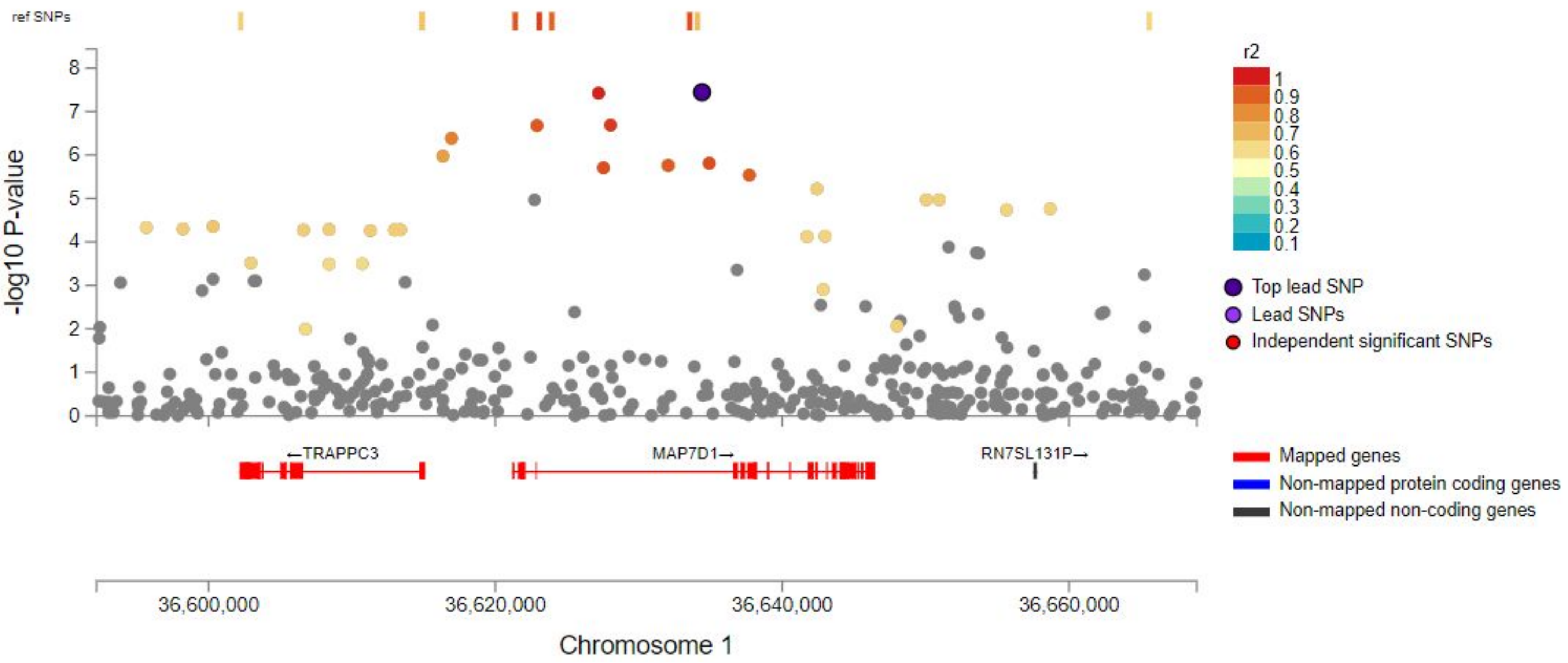


**Supplementary Figure 2a**. Locus zoom plots for early-onset MDD

| | |
|---|---|
| top lead SNP | rs7641170 |
| Chrom | 3 |
| BP | 117471895 |
| P-value | 1.503e-08 |
| #Ind. Sig. SNPs | 2 |
| #lead SNPs | 1 |
| SNPs within LD | 29 |
| GWAS SNPs within LD | 21 |

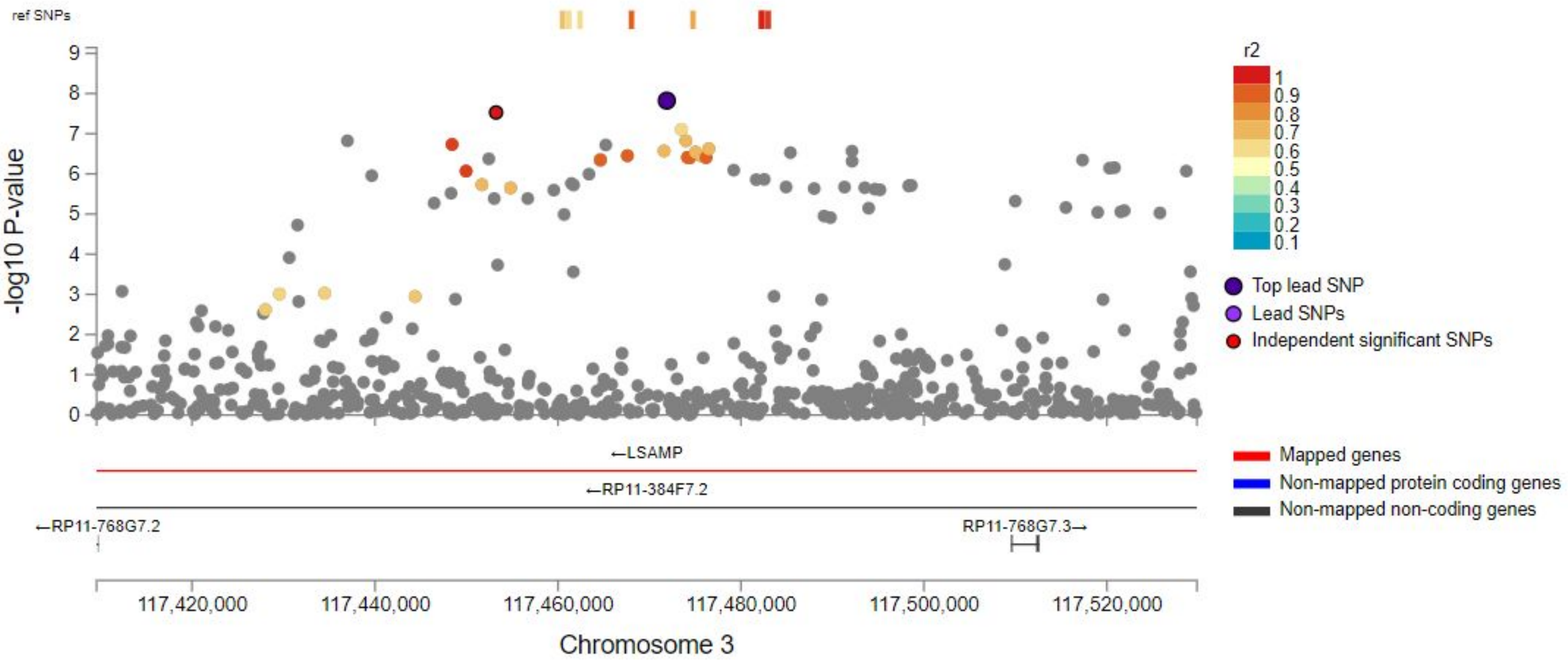


**Supplementary Figure 2b**. Locus zoom plots for early-onset MDD

| | |
|---|---|
| top lead SNP | rs73182914 |
| Chrom | 3 |
| BP | 117903064 |
| P-value | 7.105e-09 |
| #Ind. Sig. SNPs | 2 |
| #lead SNPs | 1 |
| SNPs within LD | 103 |
| GWAS SNPs within LD | 71 |

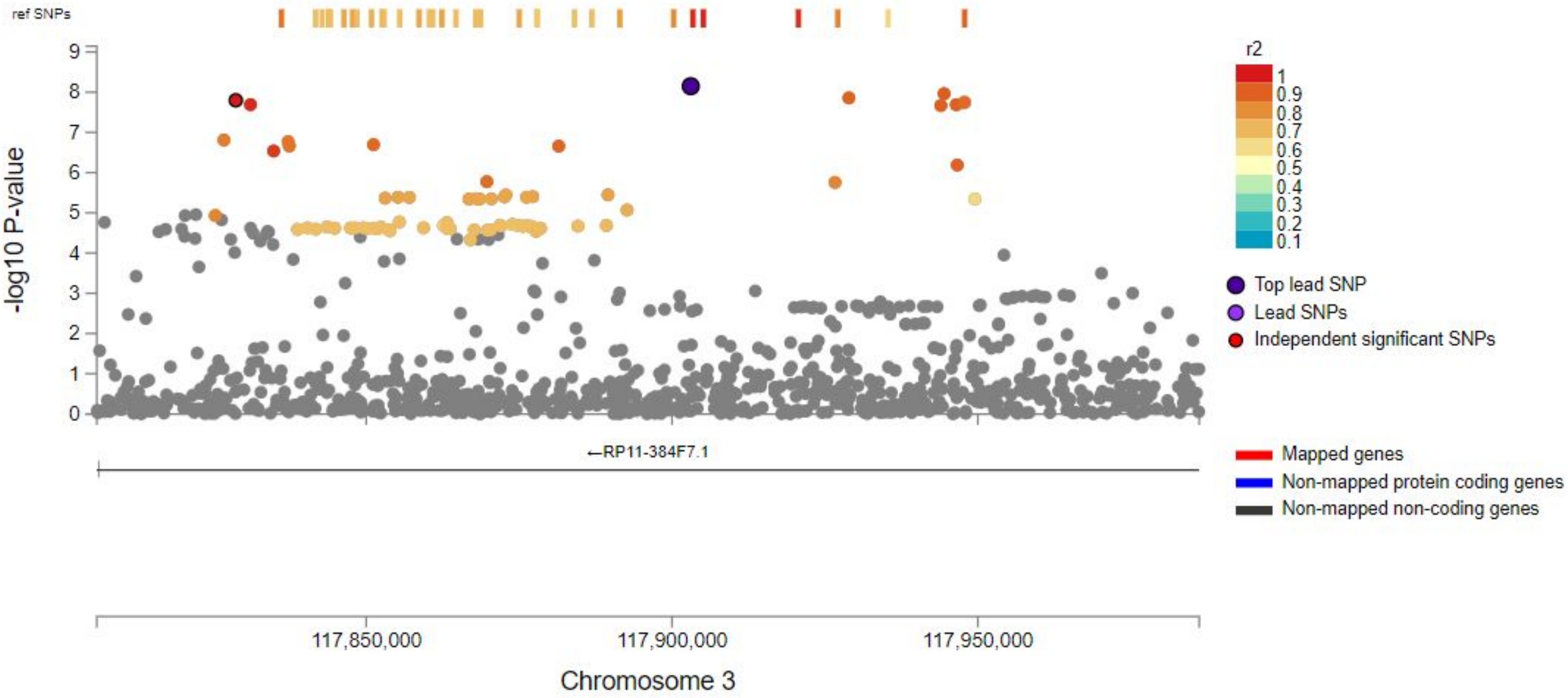


**Supplementary Figure 2c**. Locus zoom plots for early-onset MDD

| | |
|---|---|
| top lead SNP | rs6845263 |
| Chrom | 4 |
| BP | 102276682 |
| P-value | 1.041e-08 |
| #Ind. Sig. SNPs | 1 |
| #lead SNPs | 1 |
| SNPs within LD | 5 |
| GWAS SNPs within LD | 3 |

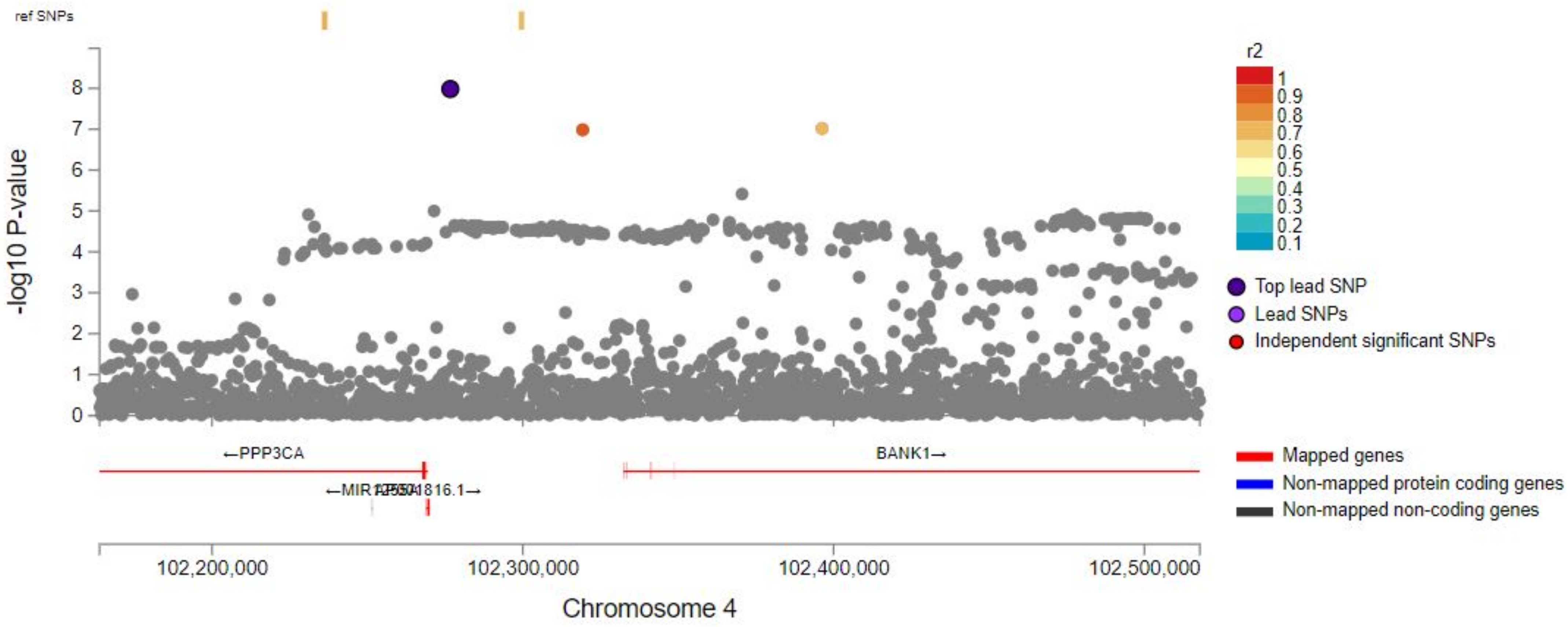


**Supplementary Figure 2d**. Locus zoom plots for early-onset MDD

| | |
|---|---|
| top lead SNP | rs2403304 |
| Chrom | 5 |
| BP | 103733250 |
| P-value | 2.891e-08 |
| #Ind. Sig. SNPs | 1 |
| #lead SNPs | 1 |
| SNPs within LD | 37 |
| GWAS SNPs within LD | 28 |

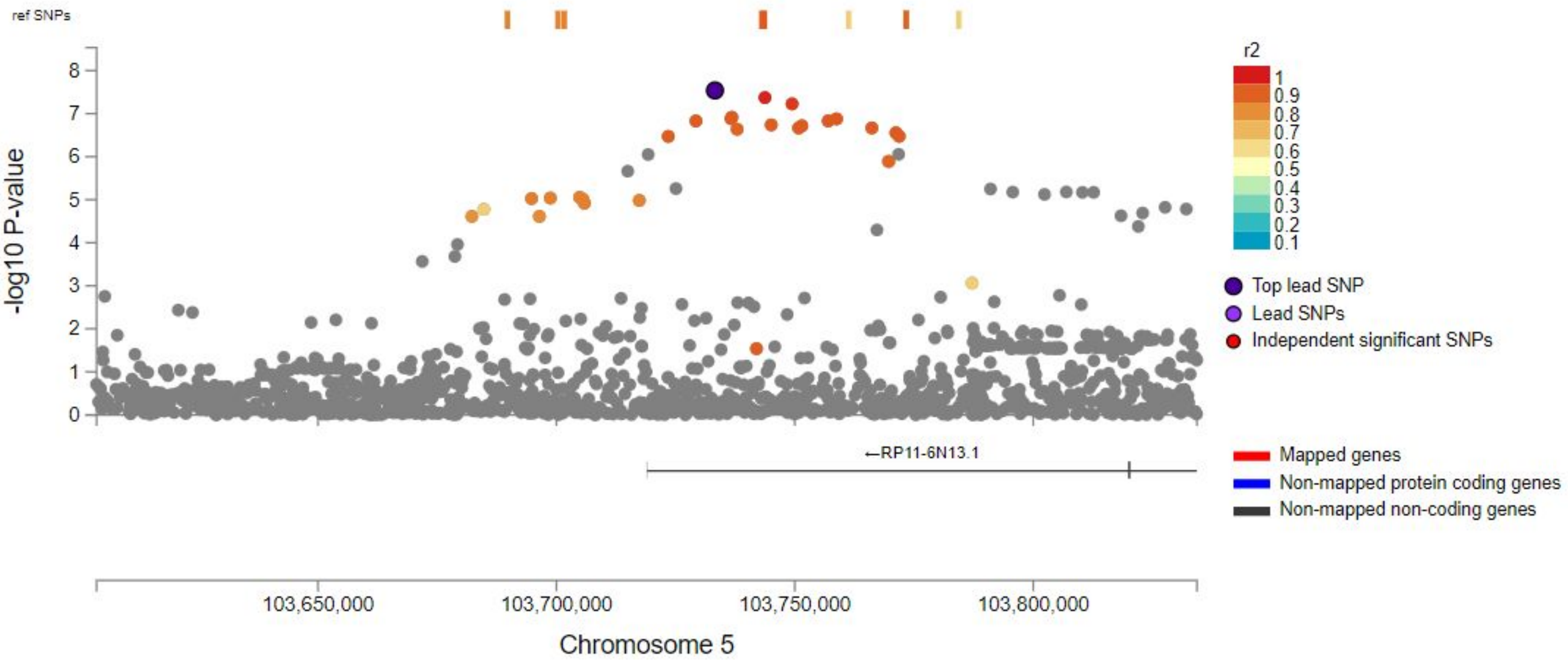


**Supplementary Figure 2e**. Locus zoom plots for early-onset MDD

| | |
|---|---|
| top lead SNP | rs3777421 |
| Chrom | 6 |
| BP | 160393386 |
| P-value | 2.574e-08 |
| #Ind. Sig. SNPs | 1 |
| #lead SNPs | 1 |
| SNPs within LD | 81 |
| GWAS SNPs within LD | 58 |

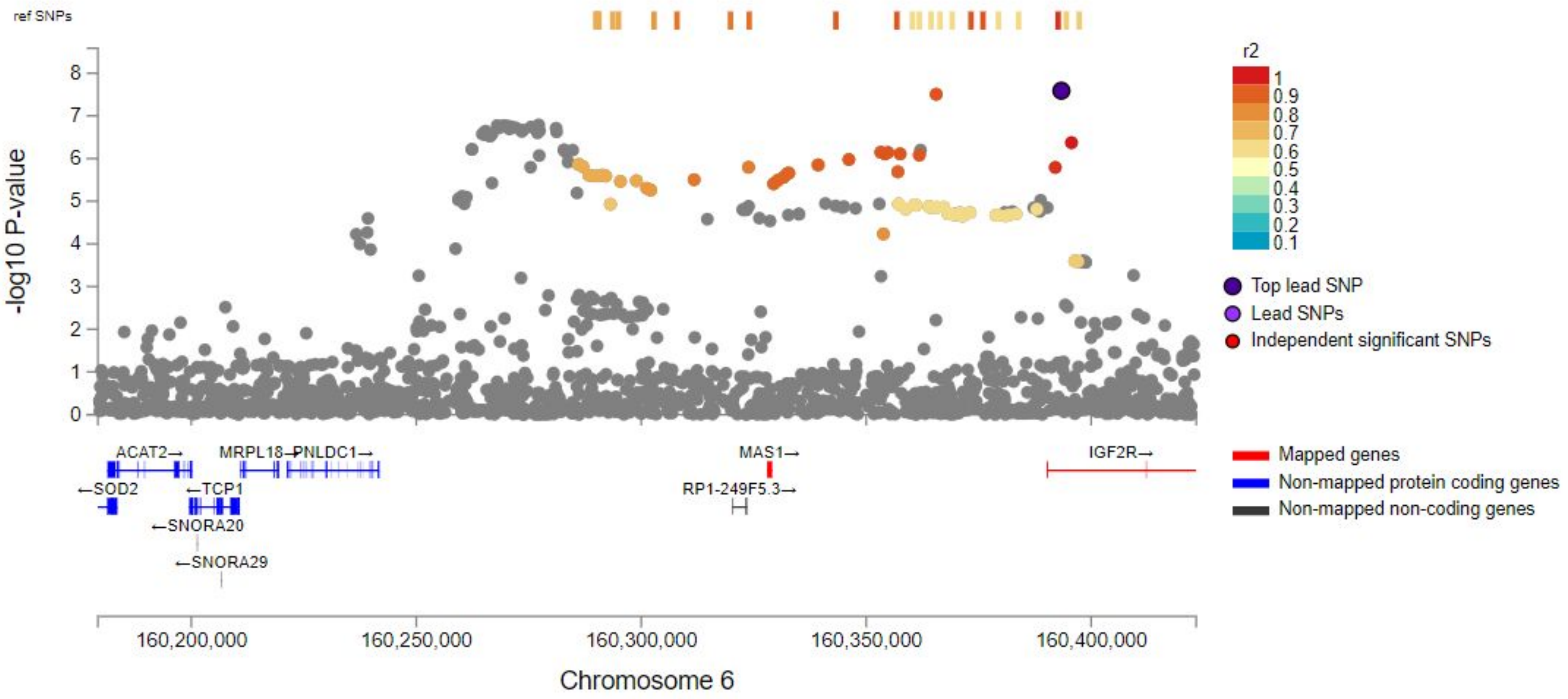


**Supplementary Figure 2f**. Locus zoom plots for early-onset MDD

| | |
|---|---|
| top lead SNP | rs11982455 |
| Chrom | 7 |
| BP | 1911458 |
| P-value | 1.503e-08 |
| #Ind. Sig. SNPs | 1 |
| #lead SNPs | 1 |
| SNPs within LD | 168 |
| GWAS SNPs within LD | 92 |

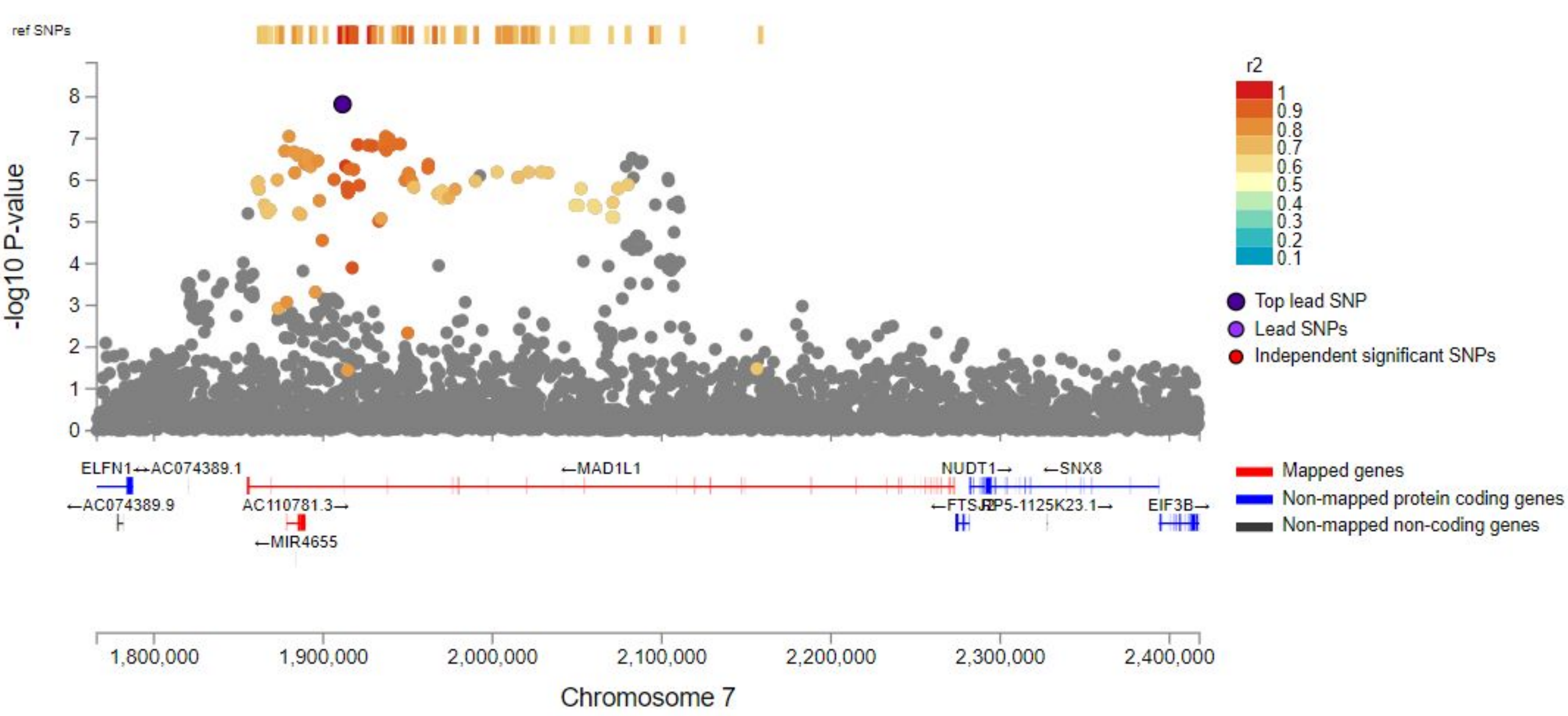


**Supplementary Figure 2g**. Locus zoom plots for early-onset MDD

| | |
|---|---|
| top lead SNP | rs57757402 |
| Chrom | 9 |
| BP | 37390411 |
| P-value | 2.541e-09 |
| #Ind. Sig. SNPs | 3 |
| #lead SNPs | 2 |
| SNPs within LD | 262 |
| GWAS SNPs within LD | 198 |

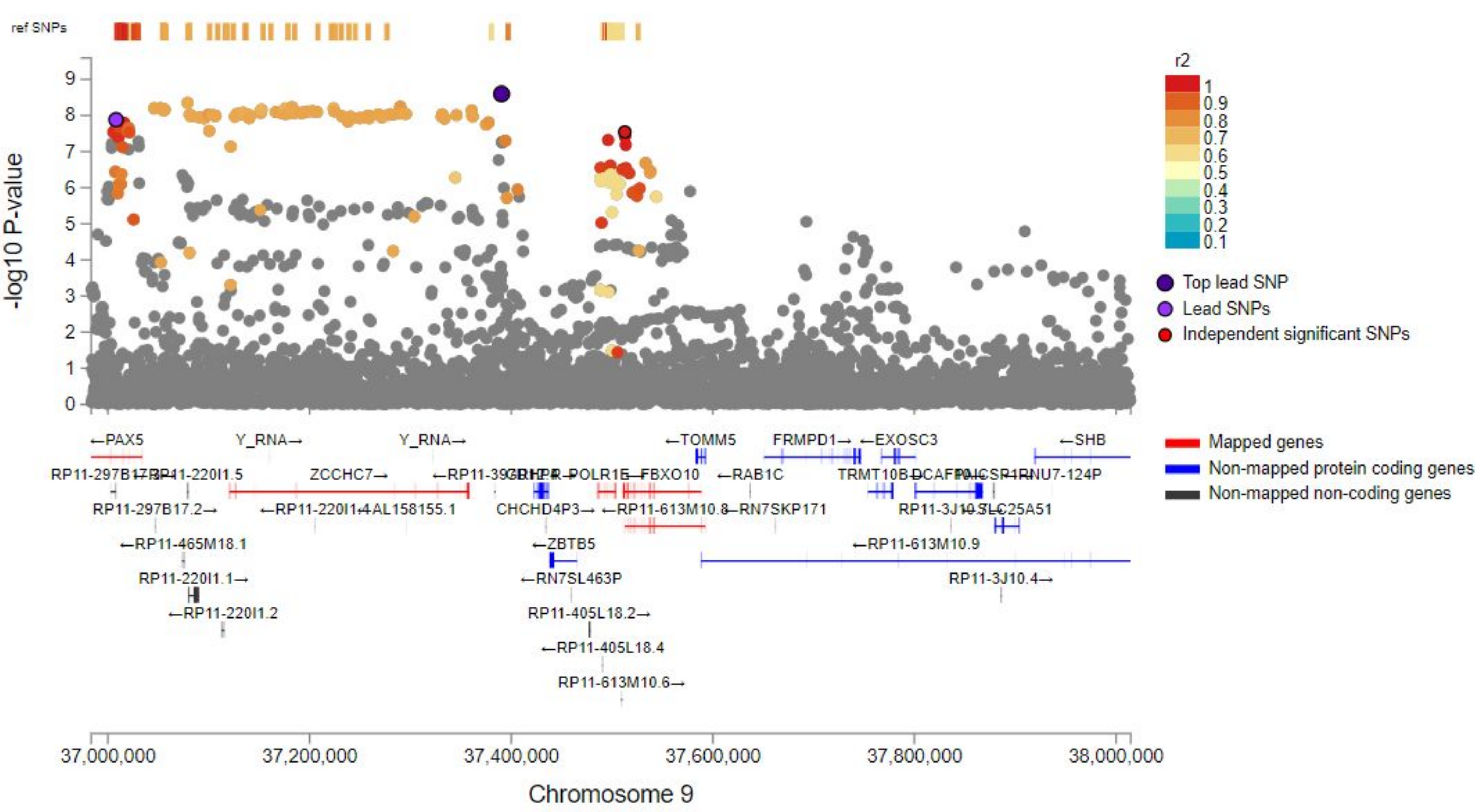


**Supplementary Figure 2h**. Locus zoom plots for early-onset MDD

| | |
|---|---|
| top lead SNP | rs11192270 |
| Chrom | 10 |
| BP | 106761616 |
| P-value | 1.077e-08 |
| #Ind. Sig. SNPs | 1 |
| #lead SNPs | 1 |
| SNPs within LD | 114 |
| GWAS SNPs within LD | 96 |

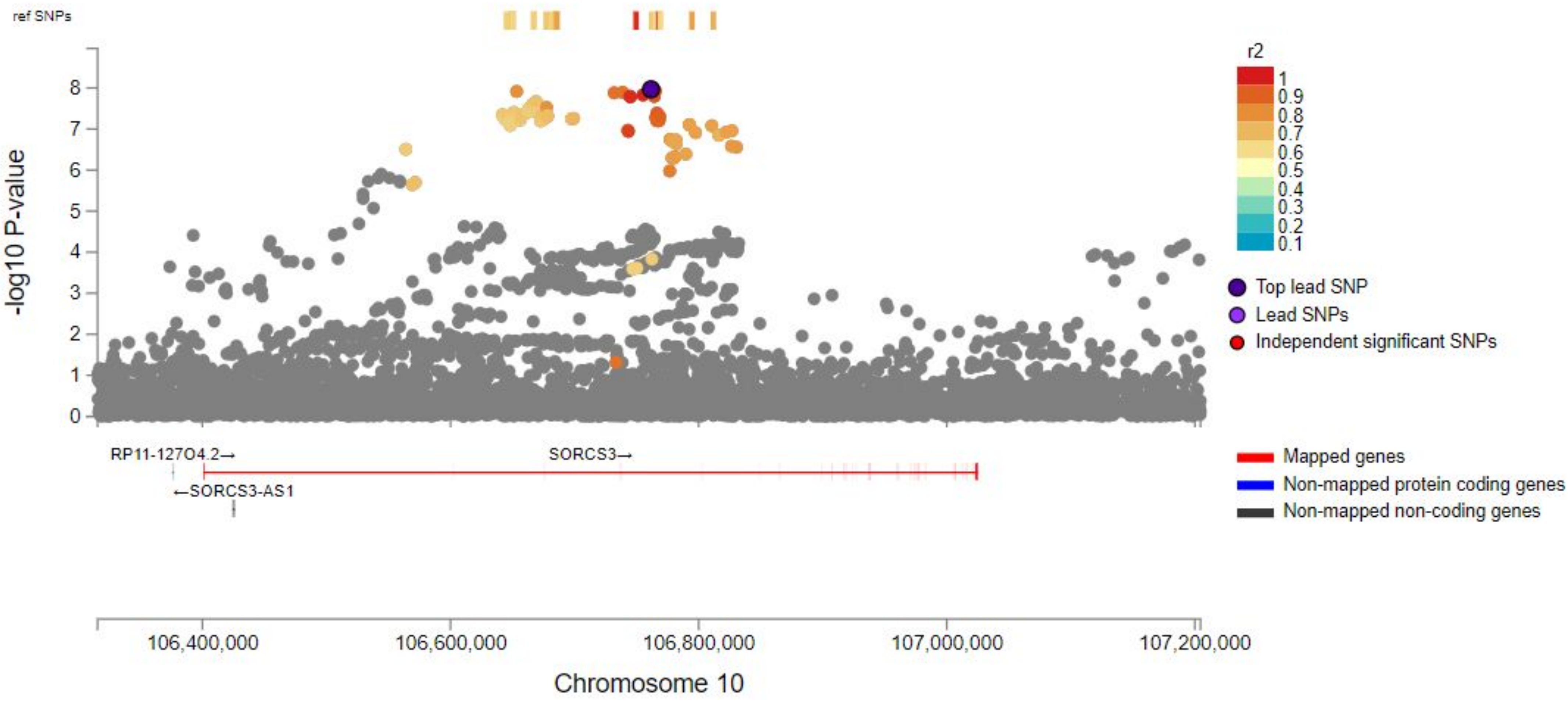


**Supplementary Figure 2i**. Locus zoom plots for early-onset MDD

| | |
|---|---|
| top lead SNP | rs7335608 |
| Chrom | 13 |
| BP | 83143774 |
| P-value | 2.377e-09 |
| #Ind. Sig. SNPs | 1 |
| #lead SNPs | 1 |
| SNPs within LD | 81 |
| GWAS SNPs within LD | 65 |

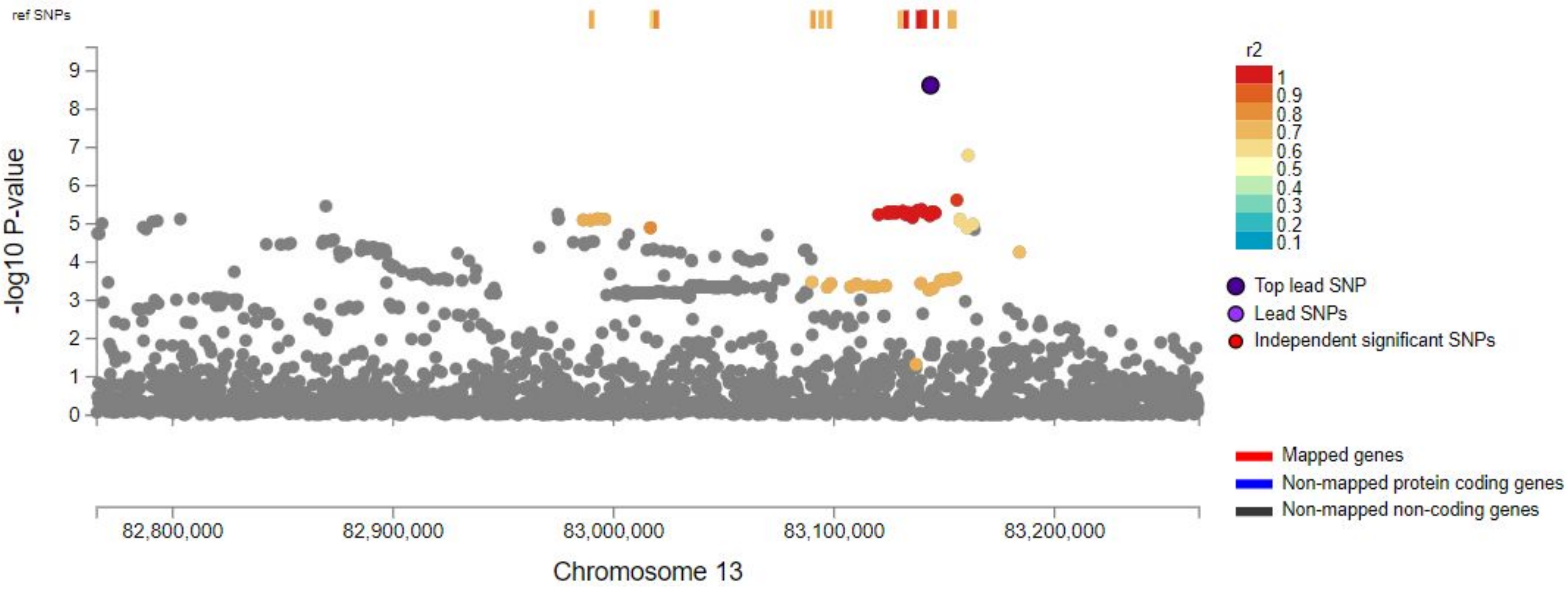


**Supplementary Figure 2j**. Locus zoom plots for early-onset MDD

| | |
|---|---|
| top lead SNP | rs12601921 |
| Chrom | 17 |
| BP | 65825354 |
| P-value | 4.63e-12 |
| #Ind. Sig. SNPs | 2 |
| #lead SNPs | 1 |
| SNPs within LD | 396 |
| GWAS SNPs within LD | 259 |

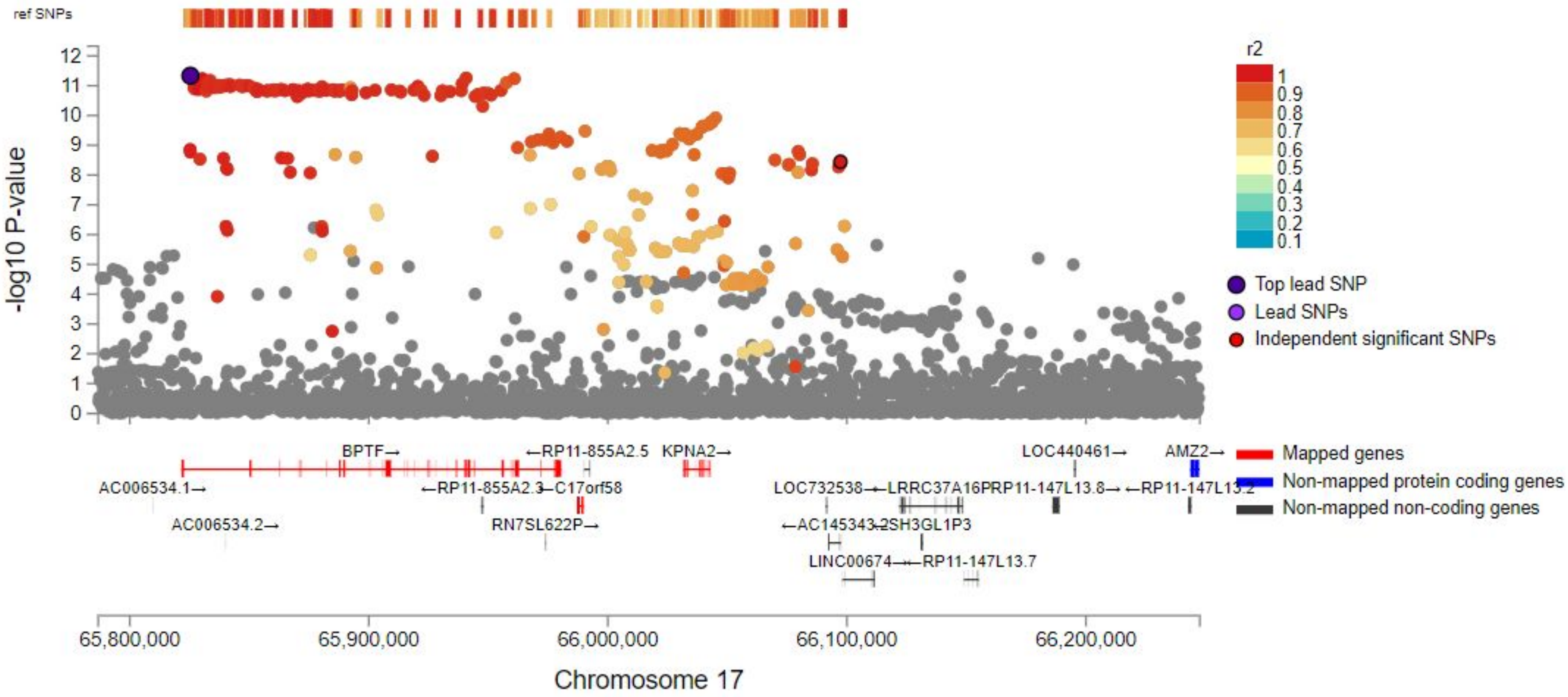


**Supplementary Figure 2k**. Locus zoom plots for early-onset MDD

| | |
|---|---|
| top lead SNP | rs72905924 |
| Chrom | 18 |
| BP | 39161790 |
| P-value | 3.911e-08 |
| #Ind. Sig. SNPs | 1 |
| #lead SNPs | 1 |
| SNPs within LD | 183 |
| GWAS SNPs within LD | 117 |

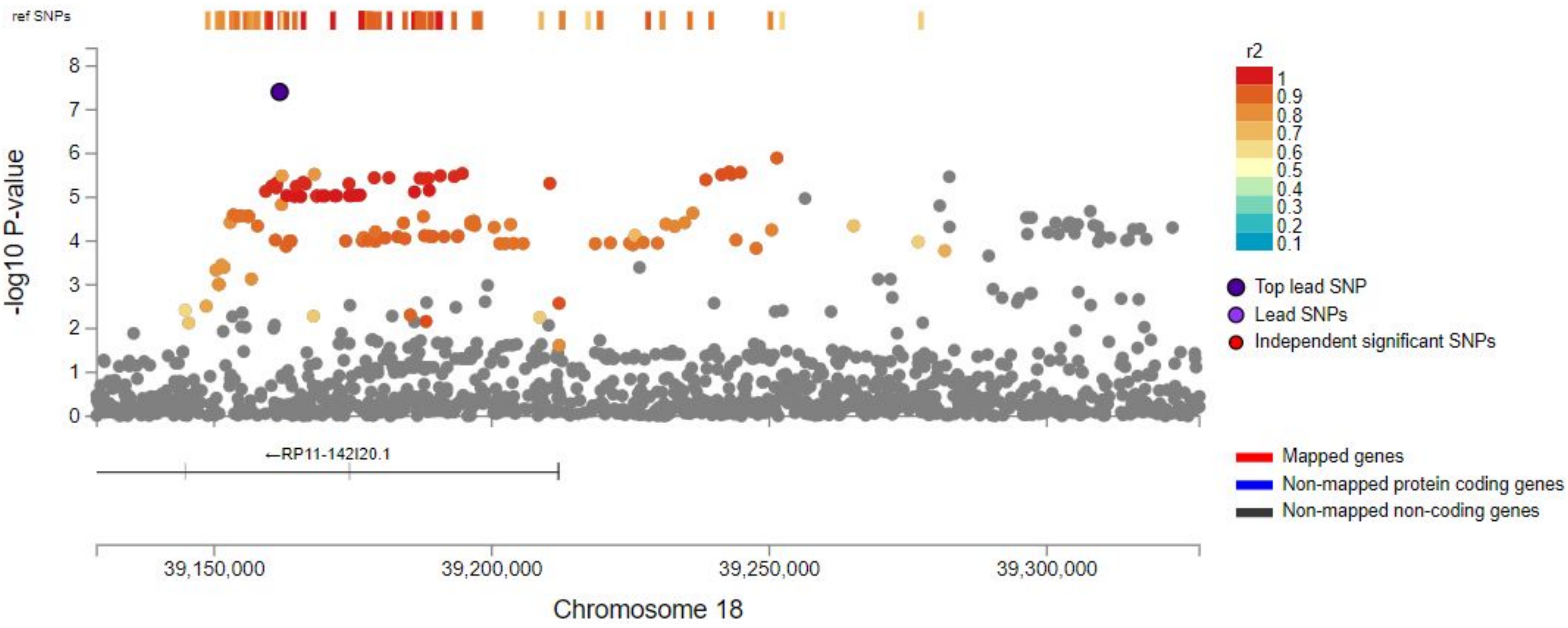


**Supplementary Figure 2l**. Locus zoom plots for early-onset MDD

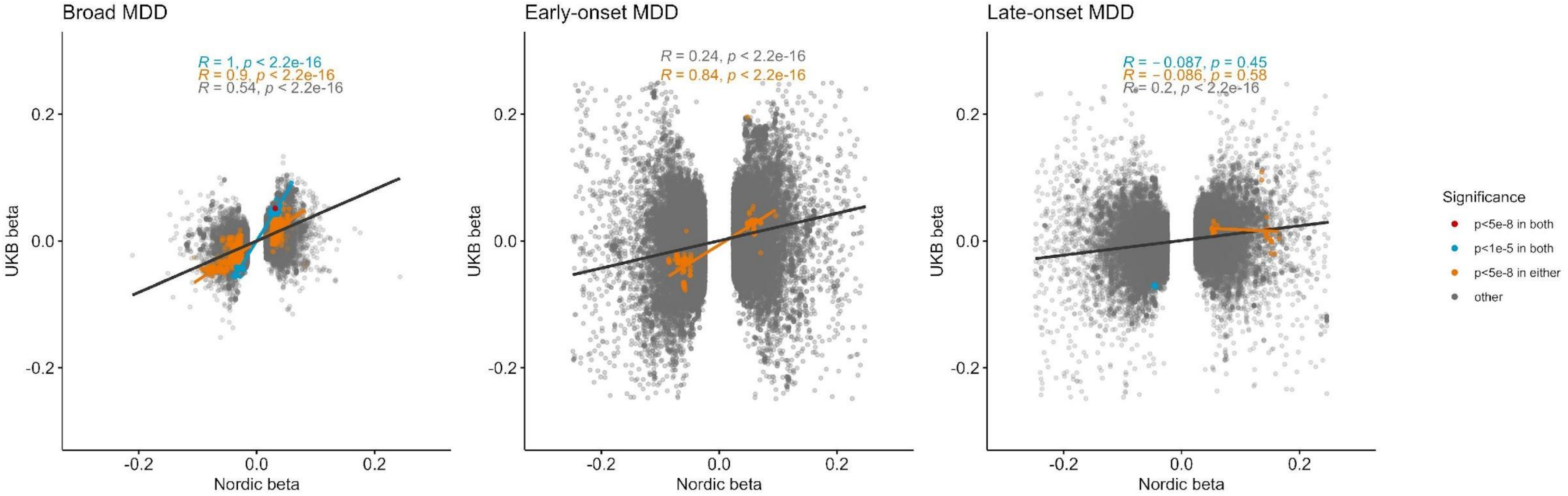


**Supplementary Figure 3a**. **SNP lookup in UK-Biobank.** Presented here are variants with a beta between -0.2 and 0.2 in UK-Biobank sample and the Nordic meta-analysis (limited range chosen for visualisation purposes). Variants that had were genome-wide significant in both analyses (p<5e-8) are shown in red, variants with suggestive p-values (p<1e-5) in both analyses are shown in blue; variants that were genome-wide significant (p<5e-8) in one of the two analyses are shown in orange. In grey are all the other variants. The Pearson correlation R between the beta-estimate in UK-Biobank and the Nordic meta-analyses is given for these subsets of variants. There were no SNPs reaching significance in both analyses for early- and late-onset MDD, and for early-onset MDD there were no variants reaching p<1e-5 in both, either.

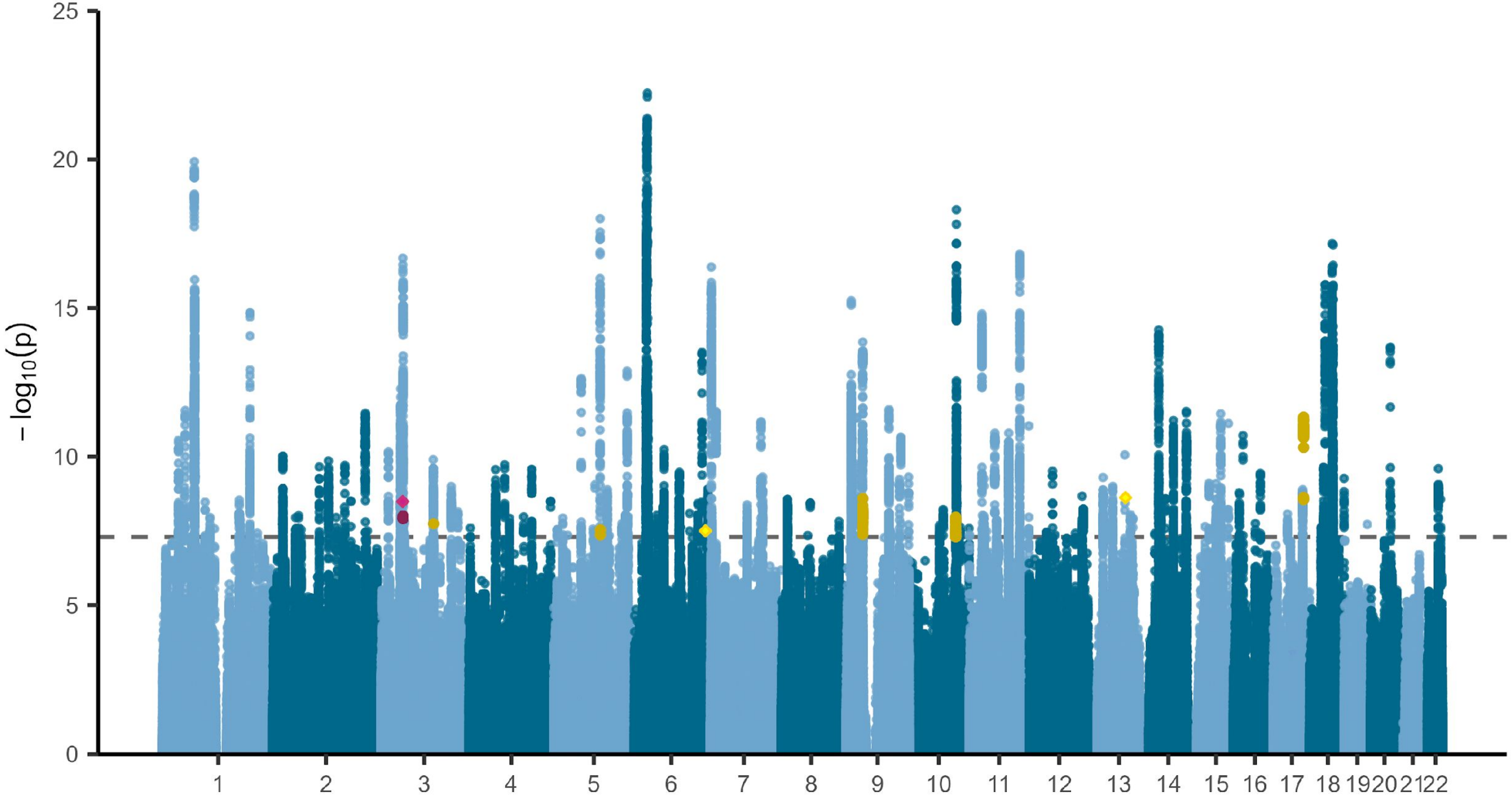


**Supplementary Figure 3b**. **SNP lookup in most recent broad MDD GWAS from Als et al., 2023** (PMID 37464041). In blue is the Manhattan plot from the results from the Als paper, with each dot representing the p-value of the association of a SNP with broad MDD. In yellow are highlighted the SNPs that were significant for early-onset MDD, and in plum the SNPs that were significant for late-onset MDD. The SNPs denoted by diamonds in a brighter shade were unique to early- and late-onset MDD and they nor their close LD partners were hits for broad MDD in the Als GWAS.

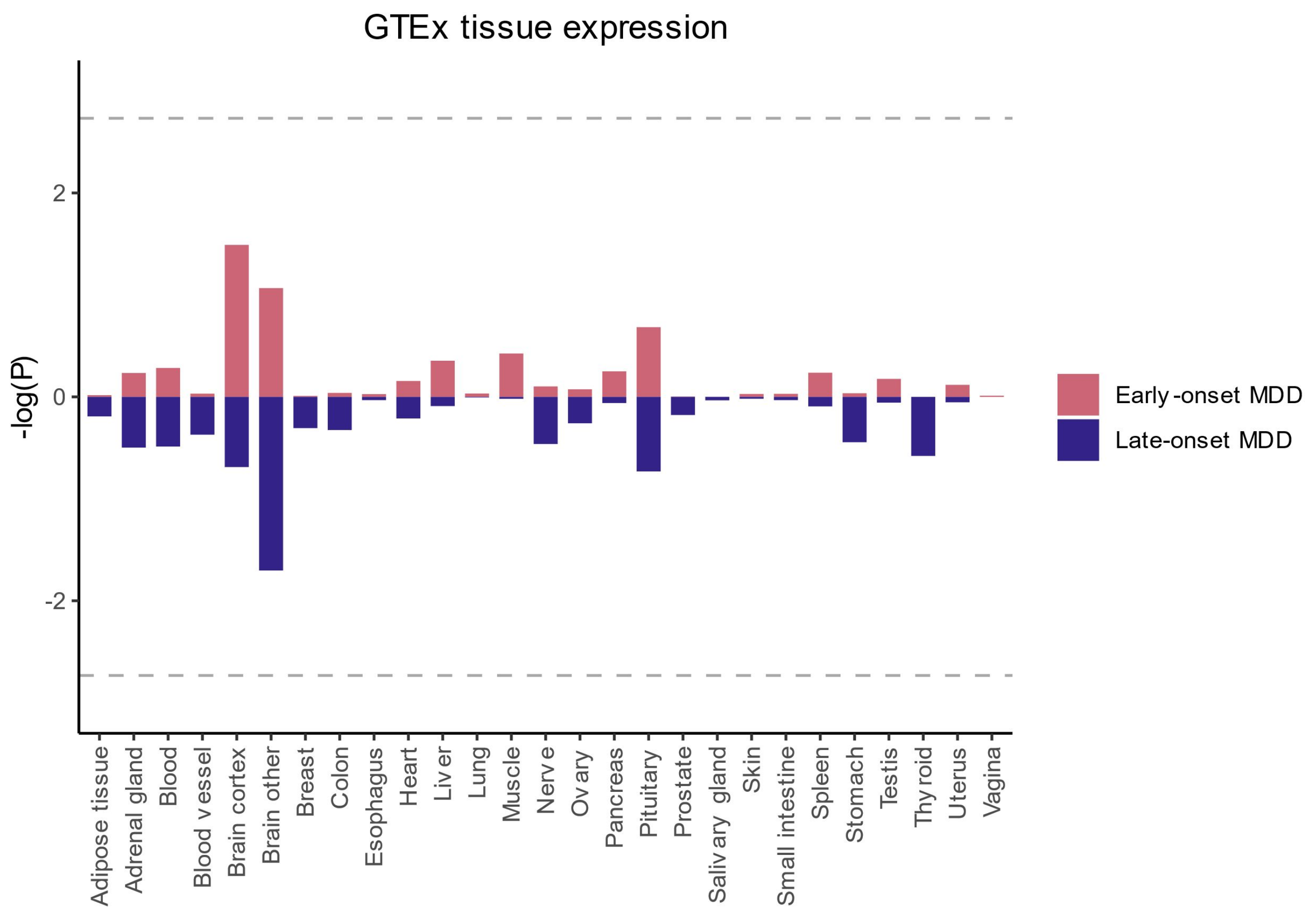


**Supplementary Figure 4a. Enrichments of SNP heritability in annotations** from GTEx tissues (27 annotations, version 8). The –log10 transformed p-value for the regression coefficient of the enrichment is presented on the y-axis, with the dashed line indicating the Bonferroni-corrected significance threshold of .05/27 tissues.

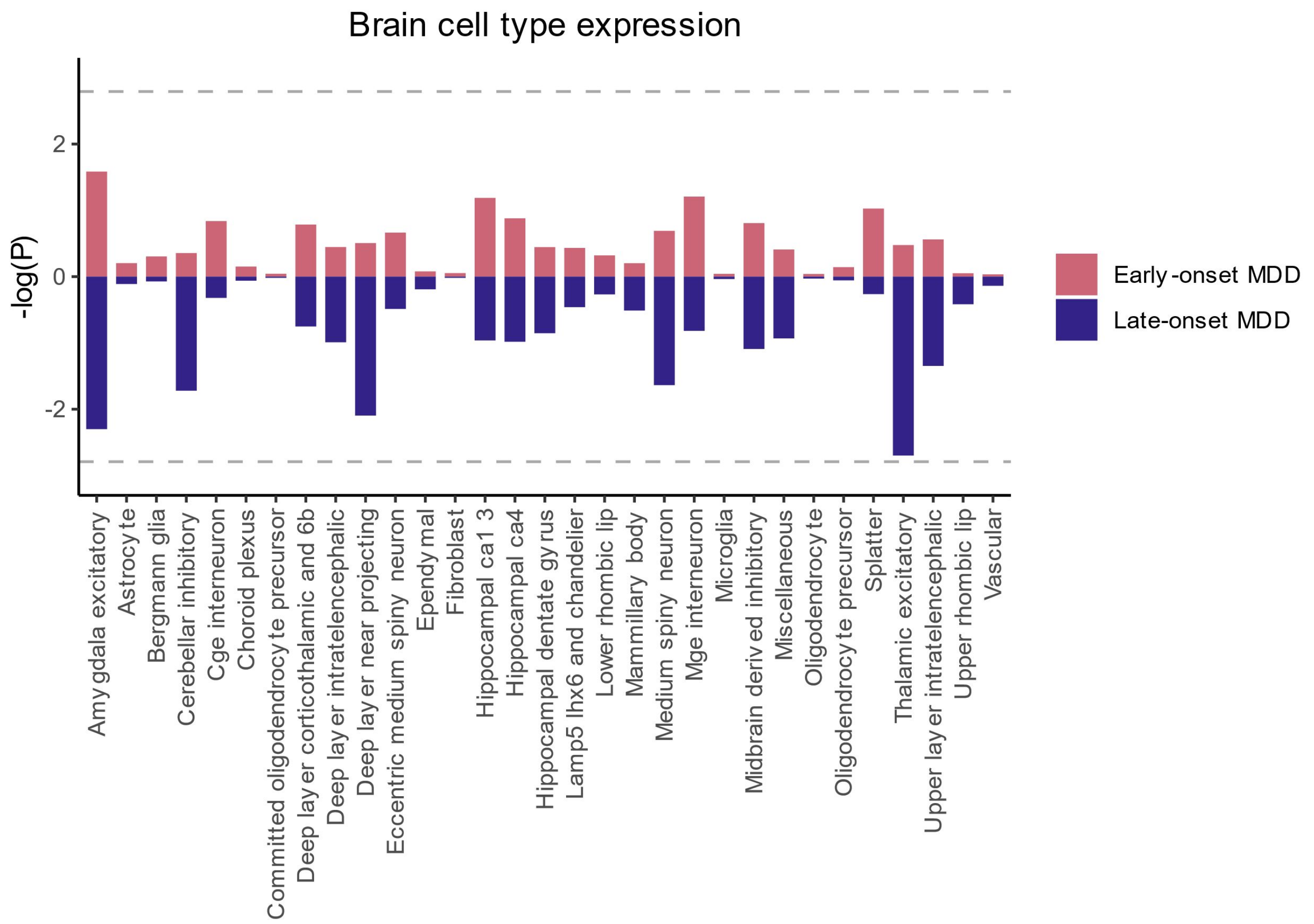


**Supplementary Figure 4b. Enrichments of SNP heritability in annotations** from human brain cell-types from the human Brain Cell Census (31 annotations). The –log10 transformed p-value for the regression coefficient of the enrichment is presented on the y-axis, with the dashed line indicating the Bonferroni-corrected significance threshold of .05/31 tissues.

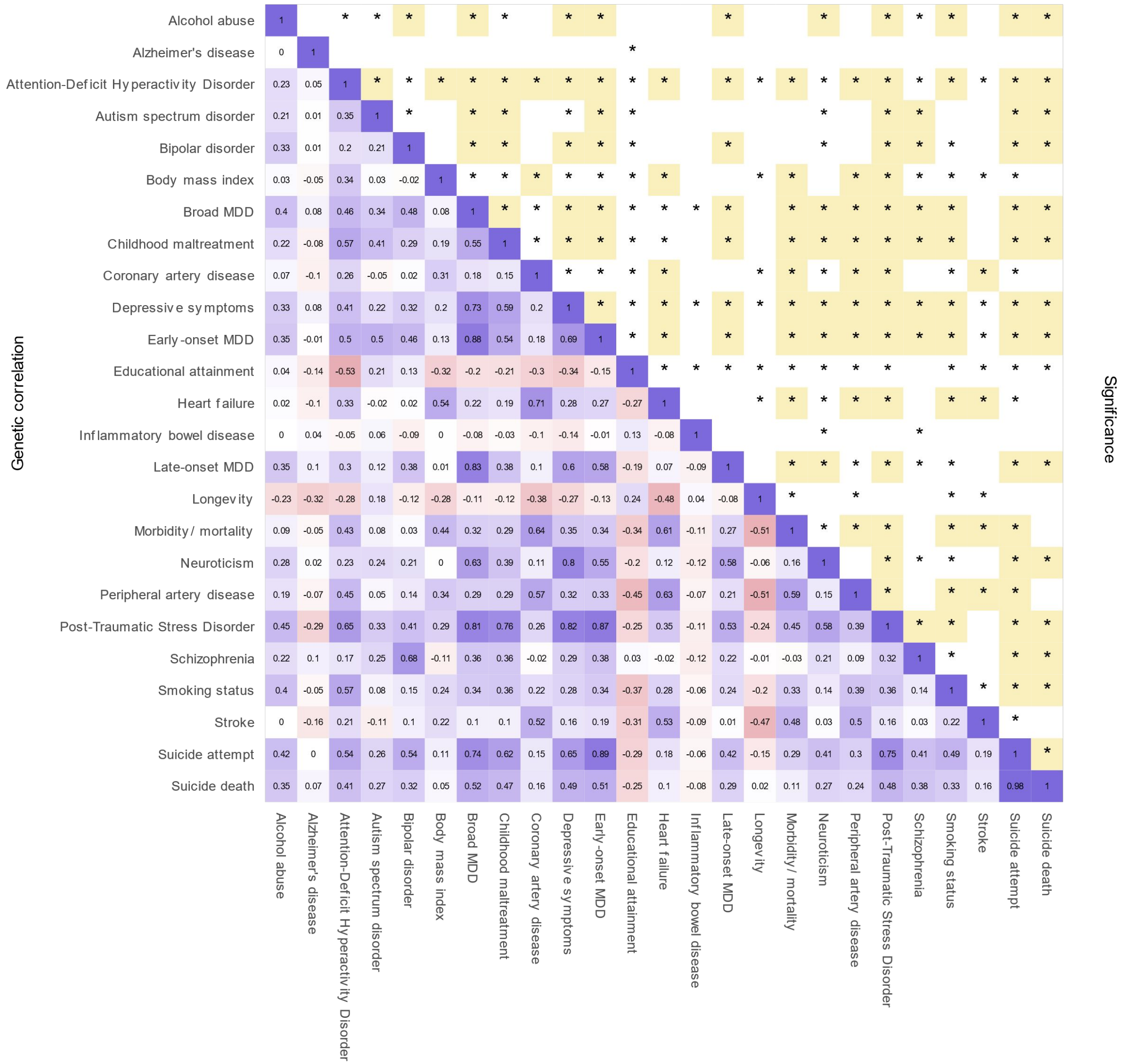


**Supplementary Figure 5**. Heatmap of genetic correlations between all traits included in the genetic correlation, Genomic SEM, and MR analyses. In the lower triangle the size of the correlation is labeled, with red indicating a negative association and blue a positive one and the darkness of the shade indicating the size of the correlation. In the upper triangle, correlations stronger than 0.25 have been highlighted, and correlations significant at $p<.002$ (.05/25, which was the number of traits included) have been denoted with an asterisk.

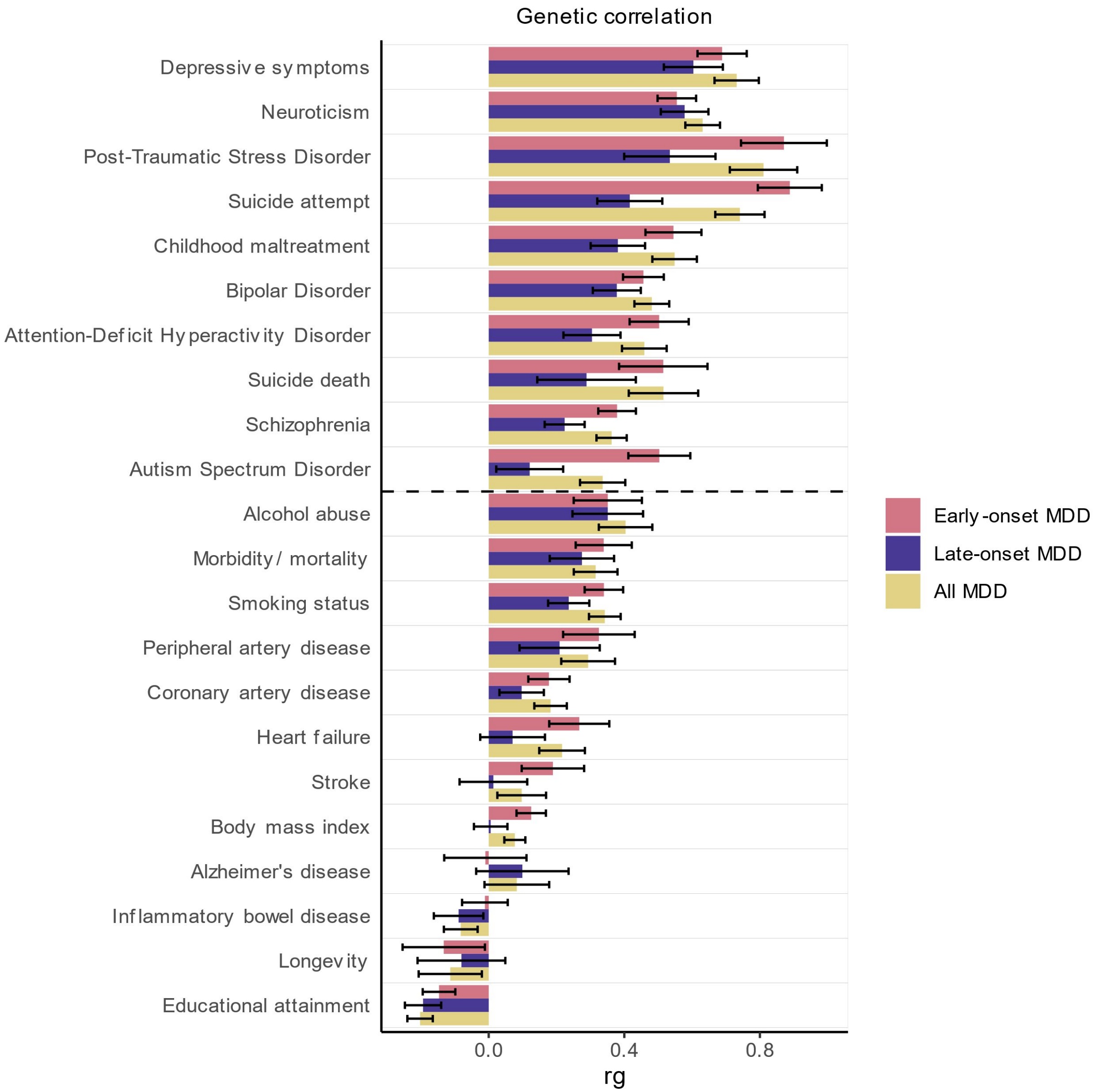


**Supplementary Figure 6**. Genetic correlation bar plot with the error bars representing 95% confidence intervals.

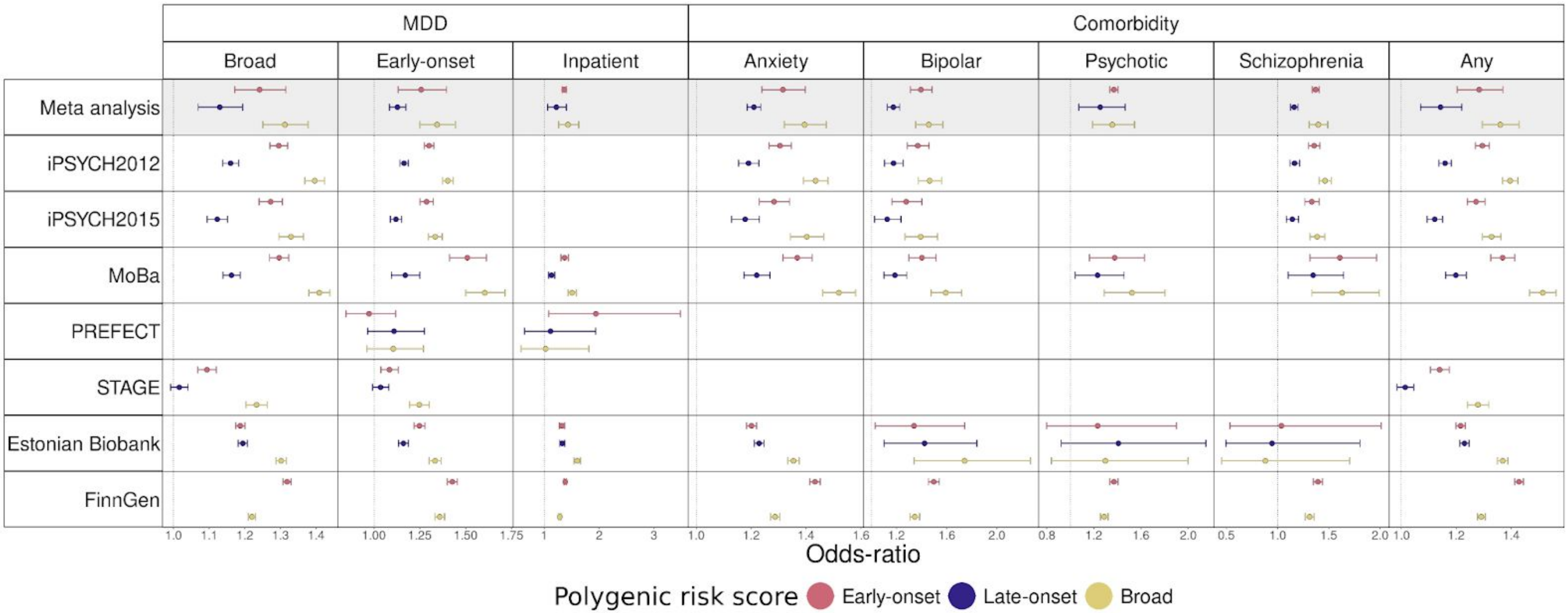


**Supplementary Figure 7. PRS for the meta-analysis and individual cohorts.** Associations between PRS for early-onset, late-onset, and broad MDD in each participating cohort and meta-analysis. Cohort and country: iPSYCH2012, iPSYCH2015 - Denmark; MoBa - Norway; PREFECT, STAGE - Sweden; FinnGen - Finland.

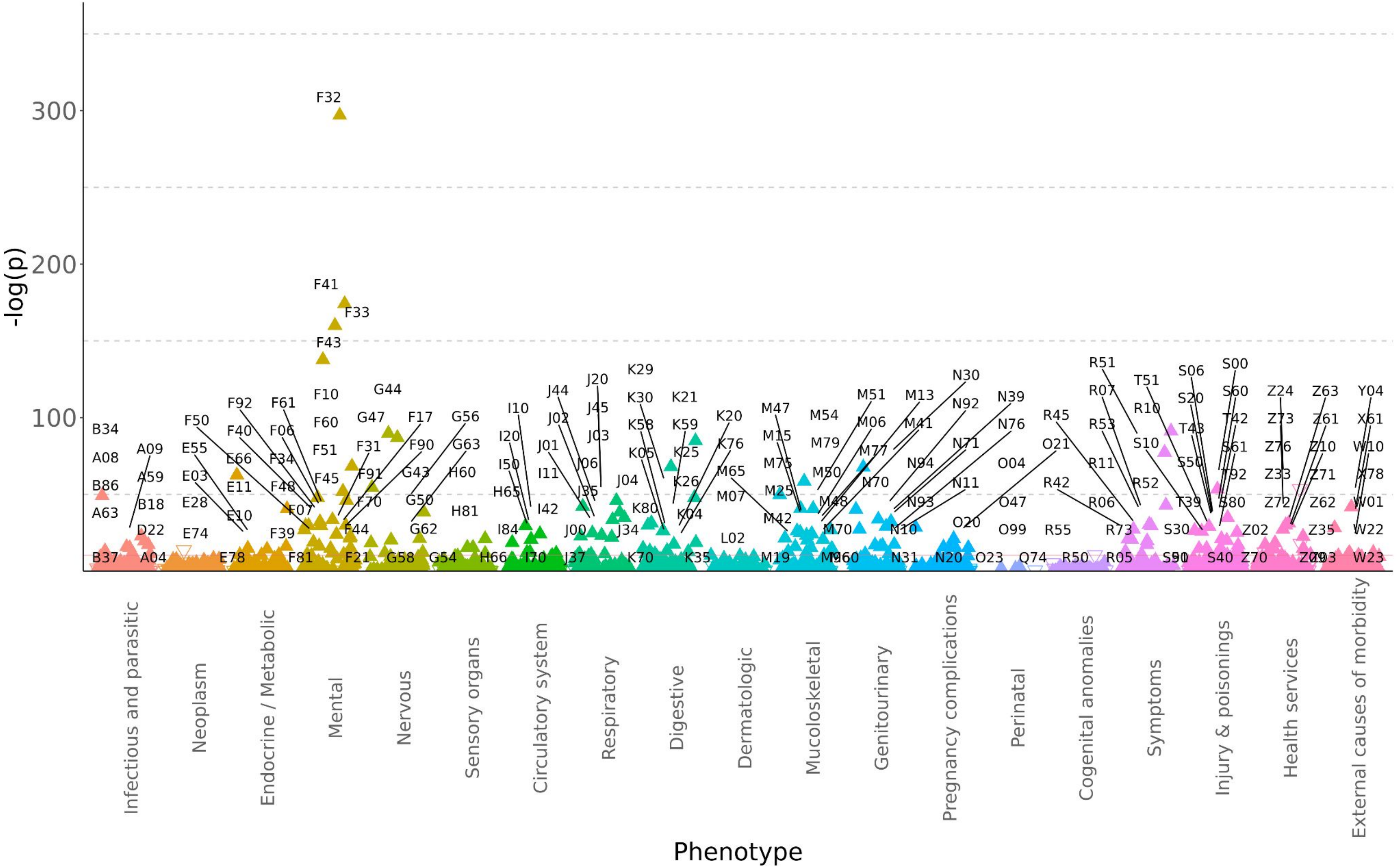


**Supplementary Figure 8a**. PGS-PheWAS results of early-onset MDD PGS. Filled triangles indicate positive association between PGS score and ICD-10 code while empty triangles negative association.

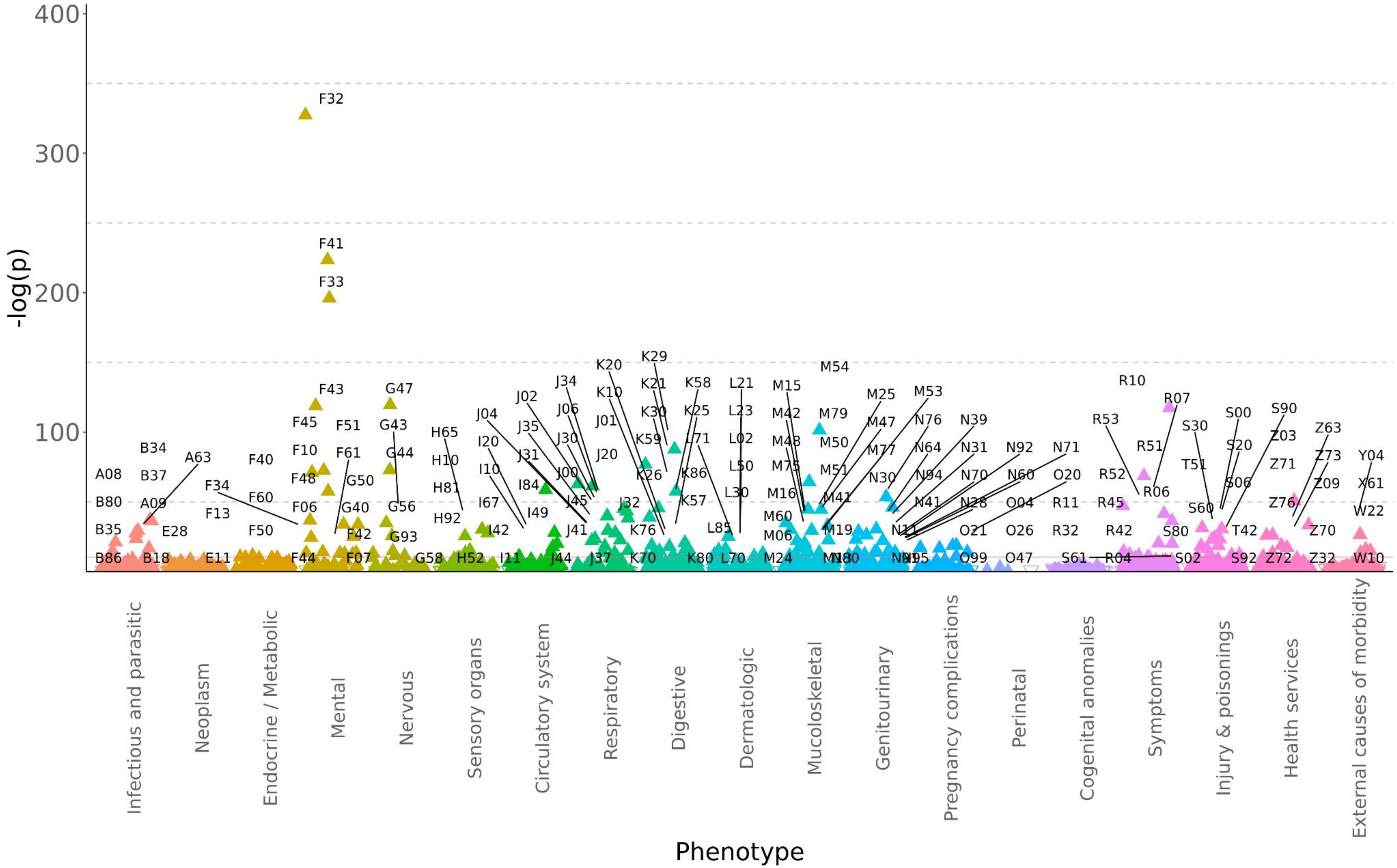


**Supplementary Figure 8b**. PGS-PheWAS results of late-onset MDD PGS. Filled triangles indicate positive association between PGS score and ICD-10 code while empty triangles negative association.
