# Supplementary material for: Genome-wide association analyses identify distinct genetic architectures for early-onset and late-onset depression
Source: Nat Genet. 2025 Nov 13;57(12):2972–9. doi: 10.1038/s41588-025-02396-8 (PMC12695632; doi:10.1038/s41588-025-02396-8)
Supplement: Supplementary file 2 — Reporting Summary [file 41588_2025_2396_MOESM2_ESM.pdf]

nature portfolio

Corresponding author(s): John Shorter, Yi Lu

Last updated by author(s): Aug 19, 2025

Reporting Summary

Nature Portfolio wishes to improve the reproducibility of the work that we publish. This form provides structure for consistency and transparency in reporting. For further information on Nature Portfolio policies, see our Editorial Policies and the Editorial Policy Checklist.

Statistics

For all statistical analyses, confirm that the following items are present in the figure legend, table legend, main text, or Methods section.

n/a	Confirmed	
☐	☒	The exact sample size (*n*) for each experimental group/condition, given as a discrete number and unit of measurement
☐	☒	A statement on whether measurements were taken from distinct samples or whether the same sample was measured repeatedly
☐	☒	The statistical test(s) used AND whether they are one- or two-sided<br>*Only common tests should be described solely by name; describe more complex techniques in the Methods section.*
☐	☒	A description of all covariates tested
☐	☒	A description of any assumptions or corrections, such as tests of normality and adjustment for multiple comparisons
☐	☒	A full description of the statistical parameters including central tendency (e.g. means) or other basic estimates (e.g. regression coefficient) AND variation (e.g. standard deviation) or associated estimates of uncertainty (e.g. confidence intervals)
☐	☒	For null hypothesis testing, the test statistic (e.g. *F*, *t*, *r*) with confidence intervals, effect sizes, degrees of freedom and *P* value noted<br>*Give P values as exact values whenever suitable.*
☒	☐	For Bayesian analysis, information on the choice of priors and Markov chain Monte Carlo settings
☐	☒	For hierarchical and complex designs, identification of the appropriate level for tests and full reporting of outcomes
☐	☒	Estimates of effect sizes (e.g. Cohen's *d*, Pearson's *r*), indicating how they were calculated

Our web collection on statistics for biologists contains articles on many of the points above.

Software and code

Policy information about availability of computer code

Data collection	This study does not involve new data collection.
Data analysis	To ensure transparent and reproducible analyses of data across sites, we developed software containers and accompanying codes for data processing and analyses (e.g., GWAS, PRS, LD score regression), and distributed the containers across the study sites. All source codes and files are publicly available via GitHub. The central code repository for the tools for GWAS and post-GWAS analyses is https://github.com/comorment/containers, while other tools and public reference data are available in the same GitHub organization.

For manuscripts utilizing custom algorithms or software that are central to the research but not yet described in published literature, software must be made available to editors and reviewers. We strongly encourage code deposition in a community repository (e.g. GitHub). See the Nature Portfolio guidelines for submitting code & software for further information.

Data

Policy information about availability of data

All manuscripts must include a data availability statement. This statement should provide the following information, where applicable:

- Accession codes, unique identifiers, or web links for publicly available datasets
- A description of any restrictions on data availability
- For clinical datasets or third party data, please ensure that the statement adheres to our policy

We have included a data availability statement as follows:
The GWAS summary statistics reported in the study are made available through Figshare: https://doi.org/10.6084/m9.figshare.27830340.

Research involving human participants, their data, or biological material

Policy information about studies with human participants or human data. See also policy information about sex, gender (identity/presentation), and sexual orientation and race, ethnicity and racism.

Reporting on sex and gender	We used biological sex in the study. It was determined based on the participants' genotypes.
Reporting on race, ethnicity, or other socially relevant groupings	We did not consider race/ethnicity in this study.<br>We determined genetic ancestry of study participants within each cohort (details are provided in Supplementary Methods).<br>We performed GWAS meta-analyses combining the Nordic cohorts of individuals with European ancestry, since we had too few samples of other ancestries to warrant separate analyses.
Population characteristics	Details are provided for each cohort in the Supplementary Methods.
Recruitment	We provide detailed descriptions of the cohorts included in this study in the Supplementary Methods.
Ethics oversight	This study was approved by the Swedish ethical review authority (Dnr 2023-03073), and each cohort was approved by the relevant institutional review boards.

Note that full information on the approval of the study protocol must also be provided in the manuscript.

Field-specific reporting

Please select the one below that is the best fit for your research. If you are not sure, read the appropriate sections before making your selection.

☒ Life sciences ☐ Behavioural & social sciences ☐ Ecological, evolutionary & environmental sciences

For a reference copy of the document with all sections, see nature.com/documents/nr-reporting-summary-flat.pdf

Life sciences study design

All studies must disclose on these points even when the disclosure is negative.

Sample size	Following harmonization of phenotypic definitions of MDD and AAO across nine cohorts from five Nordic countries (Denmark, Estonia, Finland, Norway, and Sweden) (Methods), we identified a total of 151,582 MDD cases, including 46,708 eoMDD cases with age of first diagnosis ≤ 25 years old (approximating an AAO ≤ 20-21; Methods) and 37,168 loMDD cases with age of first diagnosis ≥ 50 (approximating an AAO ≥ 44-45; Methods) (Table 1).
Data exclusions	n/a
Replication	To assess generalizability outside the Nordic cohorts, we also analyzed the UK Biobank data which relied on the self-reported age at first diagnosis, and conducted the GWAS of eoMDD and loMDD with the same age cutoffs (Fig. S1). However, considering the major differences in samples and phenotypes, we conducted primary analyses based on Nordic samples, with the UK Biobank as a replication cohort for the identified loci. Top loci were partly replicated in the UK Biobank, with one locus on chromosome 9 showing nominal significance (P<0.05); however, for the genome-wide significant loci in either sample, the correlation in their effect sizes was substantial (r=0.84 for eoMDD. Fig. S3, Tables S3-S5).
Randomization	This was a genetic association study. Allocation by genotype.
Blinding	This was a genetic association study, following observational design. So no blinding was used.

Reporting for specific materials, systems and methods

We require information from authors about some types of materials, experimental systems and methods used in many studies. Here, indicate whether each material, system or method listed is relevant to your study. If you are not sure if a list item applies to your research, read the appropriate section before selecting a response.

Materials & experimental systems

n/a	Involved in the study
☒	☐ Antibodies
☒	☐ Eukaryotic cell lines
☒	☐ Palaeontology and archaeology
☒	☐ Animals and other organisms
☒	☐ Clinical data
☒	☐ Dual use research of concern
☒	☐ Plants

Methods

n/a	Involved in the study
☒	☐ ChIP-seq
☒	☐ Flow cytometry
☒	☐ MRI-based neuroimaging

Plants

Seed stocks	N/A
Novel plant genotypes	N/A
Authentication	N/A
